# α-SNAP is expressed in mouse ovarian granulosa cells and plays a key role in folliculogenesis and female fertility

**DOI:** 10.1038/s41598-017-12292-9

**Published:** 2017-09-18

**Authors:** Alexis Arcos, Matilde de Paola, Diego Gianetti, Diego Acuña, Zahady D. Velásquez, María Paz Miró, Gabriela Toro, Bryan Hinrichsen, Rosa Iris Muñoz, Yimo Lin, Gonzalo A. Mardones, Pamela Ehrenfeld, Francisco J. Rivera, Marcela A. Michaut, Luis Federico Batiz

**Affiliations:** 1 Instituto de Anatomía, Histología y Patología, Facultad de Medicina, Universidad Austral de Chile, Valdivia, Chile; 2 Instituto de Histología y Embriología (IHEM), Universidad Nacional de Cuyo-CONICET, Mendoza, Argentina; 3 Instituto de Fisiología, Facultad de Medicina, Universidad Austral de Chile, Valdivia, Chile; 4 Center for Interdisciplinary Studies on the Nervous System (CISNe), Universidad Austral de Chile, Valdivia, Chile; 5 Institute of Molecular Regenerative Medicine, Paracelsus Medical University Salzburg, Salzburg, A-5020 Austria; 6 Spinal Cord Injury and Tissue Regeneration Center Salzburg, Paracelsus Medical University Salzburg, Salzburg, A-5020 Austria; 7 Facultad de Ciencias Exactas y Naturales, Universidad Nacional de Cuyo, Mendoza, Argentina; 8 Centro de Investigación Biomédica (CIB), Facultad de Medicina, Universidad de los Andes, Santiago, Chile; 9 Present Address: Department of Neurosurgery, Oregon Health and Science University, Portland, Oregon, USA

**Keywords:** Mechanisms of disease, Infertility

## Abstract

The balance between ovarian folliculogenesis and follicular atresia is critical for female fertility and is strictly regulated by a complex network of neuroendocrine and intra-ovarian signals. Despite the numerous functions executed by granulosa cells (GCs) in ovarian physiology, the role of multifunctional proteins able to simultaneously coordinate/modulate several cellular pathways is unclear. Soluble N-ethylmaleimide-sensitive factor (NSF) attachment protein (α-SNAP) is a multifunctional protein that participates in SNARE-mediated membrane fusion events. In addition, it regulates cell-to-cell adhesion, AMPK signaling, autophagy and apoptosis in different cell types. In this study we examined the expression pattern of α-SNAP in ovarian tissue and the consequences of α-SNAP (M105I) mutation (hyh mutation) in folliculogenesis and female fertility. Our results showed that α-SNAP protein is highly expressed in GCs and its expression is modulated by gonadotropin stimuli. On the other hand, α-SNAP-mutant mice show a reduction in α-SNAP protein levels. Moreover, increased apoptosis of GCs and follicular atresia, reduced ovulation rate, and a dramatic decline in fertility is observed in α-SNAP-mutant females. In conclusion, α-SNAP plays a critical role in the balance between follicular development and atresia. Consequently, a reduction in its expression/function (M105I mutation) causes early depletion of ovarian follicles and female subfertility.

## Introduction

In mammals, female reproductive capacity depends on a complex interplay of several regulatory mechanisms that allow the multistage process of oocyte maturation and ovulation. Oocyte development takes place in the ovarian follicle, which is composed of multiple cell types, including somatic granulosa cells (GCs) and theca cells. During folliculogenesis, primordial follicles are recruited into a growing pool through primary, preantral, and antral stages to the largest Graafian follicles that finally ovulate a mature oocyte^1^. Interestingly, only one or very few of the primordial follicles initially recruited in the growing pool are destined to ovulation and most of them undergo degenerative changes known as atresia^2^. The balance between folliculogenesis and follicular atresia is critical for female fertility and is strictly regulated by an elaborate network of neuroendocrine and paracrine signaling events and an ordered arrangement of cell-to-cell contacts within the follicle^3^. Indeed, successful oocyte survival and release at ovulation depends on an adequate gonadotropin drive that stimulates late stages of follicular growth and oocyte extrusion^4^, and proper follicular cell-to-cell contacts and local (paracrine) communication between oocyte, GCs, and theca cells^5–7^. A better understanding of the molecular machinery involved in these intra-ovarian cellular mechanisms may help to understand the control of follicular developmental dynamics during reproductive lifespan, and to define the basis underlying prevalent pathological conditions associated with female subfertility or infertility^8–10^.

Despite the vast evidence for the relevance of the balance between follicular development and follicular atresia in the ovary, the precise cellular and molecular mechanisms underlying this phenomenon remain poorly understood. Several studies indicate that cellular mechanisms regulating proliferation, autophagy, and apoptosis of GCs are involved in the initiation of follicular atresia^11–16^. Interestingly, GCs establish functional cell-to-cell contacts (gap junctions and adherens junctions) with other GCs and with the oocyte that are relevant for oocyte survival and follicular growth^17–19^. On the other hand, GCs secrete steroidal hormones (estrogens) that regulate the hypothalamic-pituitary-ovarian (HPO) axis^20^, are required for proper follicle maturation and ovulation^21^, and regulate the physiology of various organs and systems^22–26^. They also secrete, via exocytosis, a wide variety of paracrine factors including cytokines and chemokines that participate in the fine-tuning of specific intra-ovarian processes^21,27^.

Despite the numerous functions executed by GCs during folliculogenesis and ovarian physiology, little attention has been paid to the role of multifunctional proteins that can coordinate/modulate several intracellular pathways or mechanisms. α-SNAP (soluble N-ethylmaleimide-sensitive factor (NSF) attachment protein) is a multifunctional protein classically associated with intracellular membrane fusion mechanisms^28^. Basically, α-SNAP mediates, together with the ATPase NSF, the dissociation of inactive cis-SNAP receptors (SNARE) complexes, allowing subsequent rounds of membrane fusion events^29^. Thus, α-SNAP is involved in many different secretory processes^30–36^. Notably, in addition to these canonical SNARE-mediated/NSF-dependent functions of α-SNAP, novel non-canonical SNARE/NSF-independent functions have recently been demonstrated. Thus, α-SNAP regulates, in an NSF-independent fashion, cellular processes such as cell-cell adhesion^37^, cell-extracellular matrix adhesion^38^, AMPK signaling^39^, calcium homeostasis/signaling, autophagy^40^, and apoptosis^41^.

The function of α-SNAP in mammalian organisms has started to be elucidated in the last decade by using a spontaneous mutant mouse for α-SNAP. In fact, knocking out the mouse α-SNAP gene (Napa) results in early embryonic lethality^42^. A spontaneous missense mutation in the Napa gene that arose spontaneously in the C57BL/10 J mouse strain provokes a recessive inheritable disease known as hyh (hydrocephalus with hop gait)^42–45^. The hyh mutation causes the substitution of a highly conserved methionine at amino acid residue 105 for an isoleucine (M105I)^42,43^. Animals homozygous for the hyh mutation (Napa^hyh/hyh^) present a well-characterized neuropathological phenotype^46–49^. Remarkably, the severity of the hyh phenotype is heterogeneous among mutant animals, suggesting that other genes and/or environmental factors may modulate the phenotypic outcome of the hyh mutation^48^. Consequently, mice showing a rapidly progressive (RP) phenotype die before weaning, while those showing a slowly progressive (SP) phenotype survive longer, allowing researchers to investigate the role of α-SNAP in adulthood^48^. Using this model, we have previously shown that α-SNAP is expressed in adult mouse testis/sperm and that its function is critical for male fertility^50^. Conversely, the expression of α-SNAP in ovarian tissue and its role in follicular development and female fertility remain unknown.

Here, we studied the expression profile of α-SNAP in ovarian tissue and granulosa cells at different postnatal stages and after gonadotropin stimuli. We also studied the consequences of hyh (M105I) mutation of α-SNAP in ovarian folliculogenesis and female fertility.

## Results

### α-SNAP is expressed in granulosa cells (GCs) of ovarian follicles

**Figure 1:**
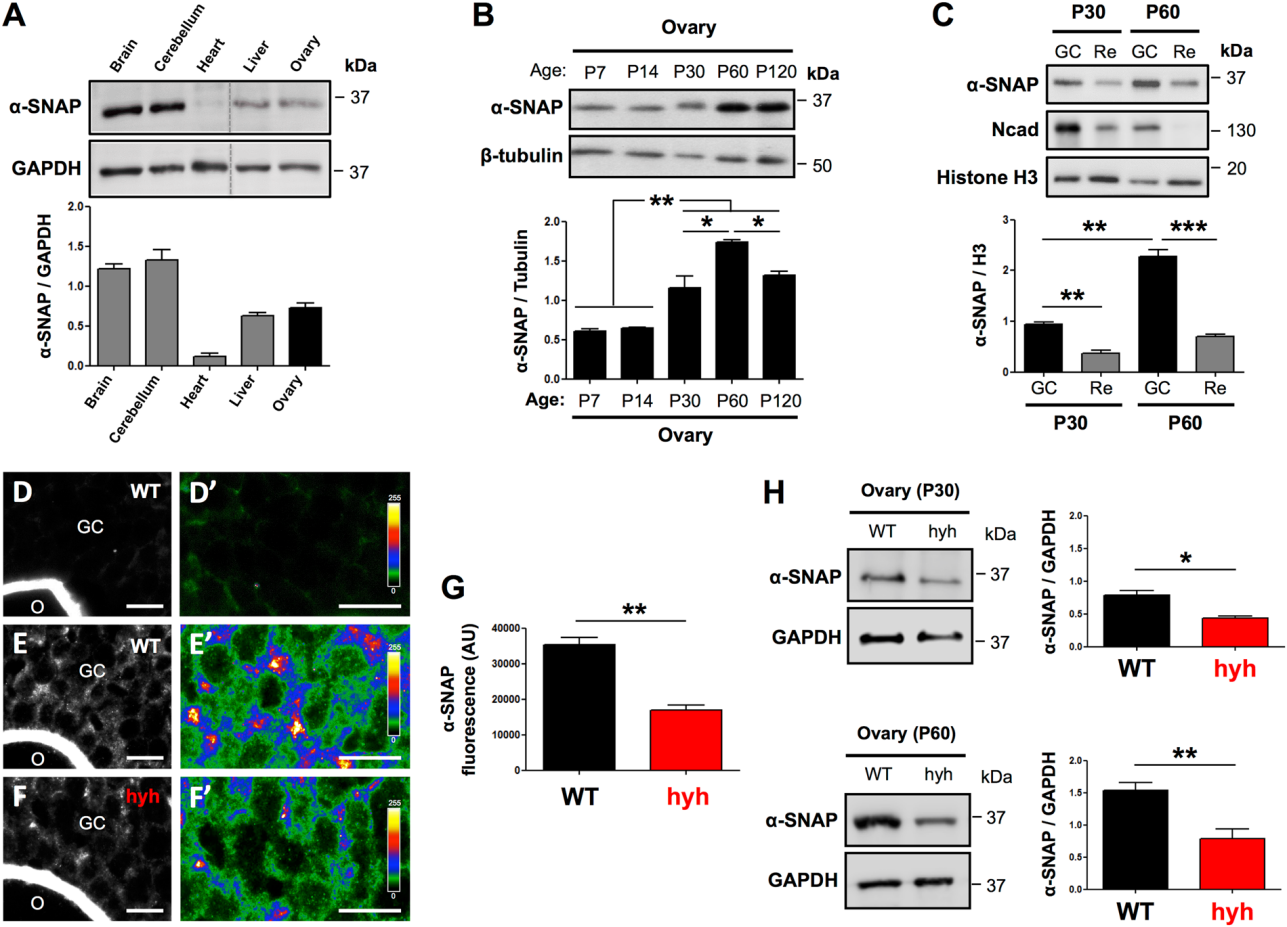
α-SNAP expression in mouse ovarian tissue and granulosa cells (GCs) of wild type (WT) and α-SNAP mutant (hyh) females. (**A**) Western blot analysis of α-SNAP in postpubertal (postnatal day 60; P60) WT female mouse tissue extracts. GAPDH levels serve as loading control. Cropped blots (dotted lines) are displayed. Full-length blots including other tissue extracts are included in the Supplementary Figures file online (see Supplementary Figure S1). (**B**) Western blot analysis of α-SNAP in prepubertal (P7 and P14), peripubertal (P30), and postpubertal (P60 and P120) ovary extracts. β-tubulin levels serve as loading control. (**C**) Western blot analysis of α-SNAP in purified GCs and ovary remnants depleted of GCs (Re) at P30 and P60. N-cadherin (Ncad) was used as a marker of GC enrichment (see Supplementary Information) and Histone H3 was used as loading control. (**A**–**C)** Bars represent mean ± SEM of densitometric analyses (n = 3 or 4 independent experiments). (**D–F**) α-SNAP immunolabeling in P60 WT (**E**) and mutant hyh (**F**) ovarian follicles. Omission of the primary antibody was used as a negative control (**D**). Note the expression of α-SNAP in GCs. The expression of α-SNAP in the oocyte (**O**) cannot be established because of a non-specific fluorescent signal detected in the oocyte-zona pellucida region. (**D**) Scale bars, 10 μm. (**D’–F’**) Magnifications of GC regions in ovarian follicles. Images were pseudocolored using the lookup table shown at the right of the figures to highlight the differences in fluorescence intensity between WT (**E’**) and mutant hyh (**F’**) samples. Scale bars, 10 μm. (**G**) Quantification of α-SNAP immunofluorescence intensity in WT (black bar) and hyh (red bar) GCs. Bars represent mean ± SEM of 4 independent experiments. (**H**) Western blot images and densitometric analysis of α-SNAP in WT and hyh ovaries at P30 and P60. GAPDH was used as loading control. Note the hypomorphism of α-SNAP in hyh samples at both developmental stages. Bars represent mean ± SEM of densitometric analyses (n = 3 independent experiments). *p < 0.05; **p < 0.01; ***p < 0.001 (ANOVA with Tukey’s post hoc test or Student’s t-test).

To gain insights into the biological relevance of α-SNAP in ovarian physiology and female fertility, we first examined the expression of α-SNAP protein in multiple wild type (WT) mouse tissue and ovarian extracts. Western blot analysis showed that α-SNAP protein is readily detected in the ovary of adult mice (Fig. 1A). We then assessed the expression of α-SNAP protein in prepubertal (P7, P14), peripubertal (P30) and postpubertal (P60, P120) stages. Interestingly, we found that expression of α-SNAP protein increased at P30 and reached a maximum at early postpubertal stages (P60) (Fig. 1B). Considering the fact that before puberty most follicles are restricted to primordial/primary/preantral stages and that after puberty the number of early antral/antral follicles drastically increases, these data suggest that ovarian α-SNAP protein levels may be related to follicle stage development and/or maturation of GCs. Thus, the expression of α-SNAP protein was tested in isolated ovarian GCs obtained from P30 and P60 ovaries (Fig. 1C). N-cadherin was used as a marker for GC enrichment since it is highly and preferentially expressed in GCs at different follicular stages^18,51^ (Fig. 1C; see Supplementary Fig. S2 and Supplementary Information for details). We found that α-SNAP is enriched in GCs and, conversely, it is relatively less abundant in ovarian remnants after GCs removal (Fig. 1C). Remarkably, the levels of α-SNAP protein were higher in GCs obtained from P60 ovaries relative to GCs derived from P30 ovaries (Fig. 1C). Supporting these results, immunofluorescence staining of P60 WT ovary sections showed that α-SNAP protein is highly expressed in GCs of growing follicles (Fig. 1E–E’). Even though we have recently described the presence of α-SNAP protein in the cortical region of isolated mouse oocytes^34^, we were not able to define by immunofluorescence staining the expression of α-SNAP in oocytes of ovary sections because of the presence of a non-specific fluorescent signal at the oocyte/zona pellucida region (Fig. 1D–D’). Subsequently, we checked the expression of α-SNAP in hyh mutant ovaries. It has been previously described that α-SNAP levels are reduced (hypomorphism) in the brain and the male reproductive tract of hyh mice^45,50^. Similarly, a significant difference in α-SNAP immunofluorescence intensity between hyh and WT GCs was observed (Fig. 1G; compare Fig. 1F–F’ to Fig. 1E–E’). In agreement with these results, Western blot analysis revealed that the amount of α-SNAP was reduced in ovaries of hyh compared with WT mice, a finding that was more evident in postpubertal (P60) than in peripubertal (P30) stages (Fig. 1H). This result was not limited to the ovaries as other tissues obtained from P60 hyh mutant females also showed hypomorphism (see Supplementary Fig. S3). To discard any irregularity regarding the immunoreactivity of the antibody with the mutant (M105I) α-SNAP protein, we tested the antibody binding by western blot assays loading different amounts of WT and M105I purified recombinant proteins (see Supplementary Fig. S4). The results revealed that the antibody recognizes with the same immunoreactivity both, WT and mutant M105I proteins (see Supplementary Fig. S5).

### Ovarian α-SNAP protein levels are modulated by gonadotropins

**Figure 2:**
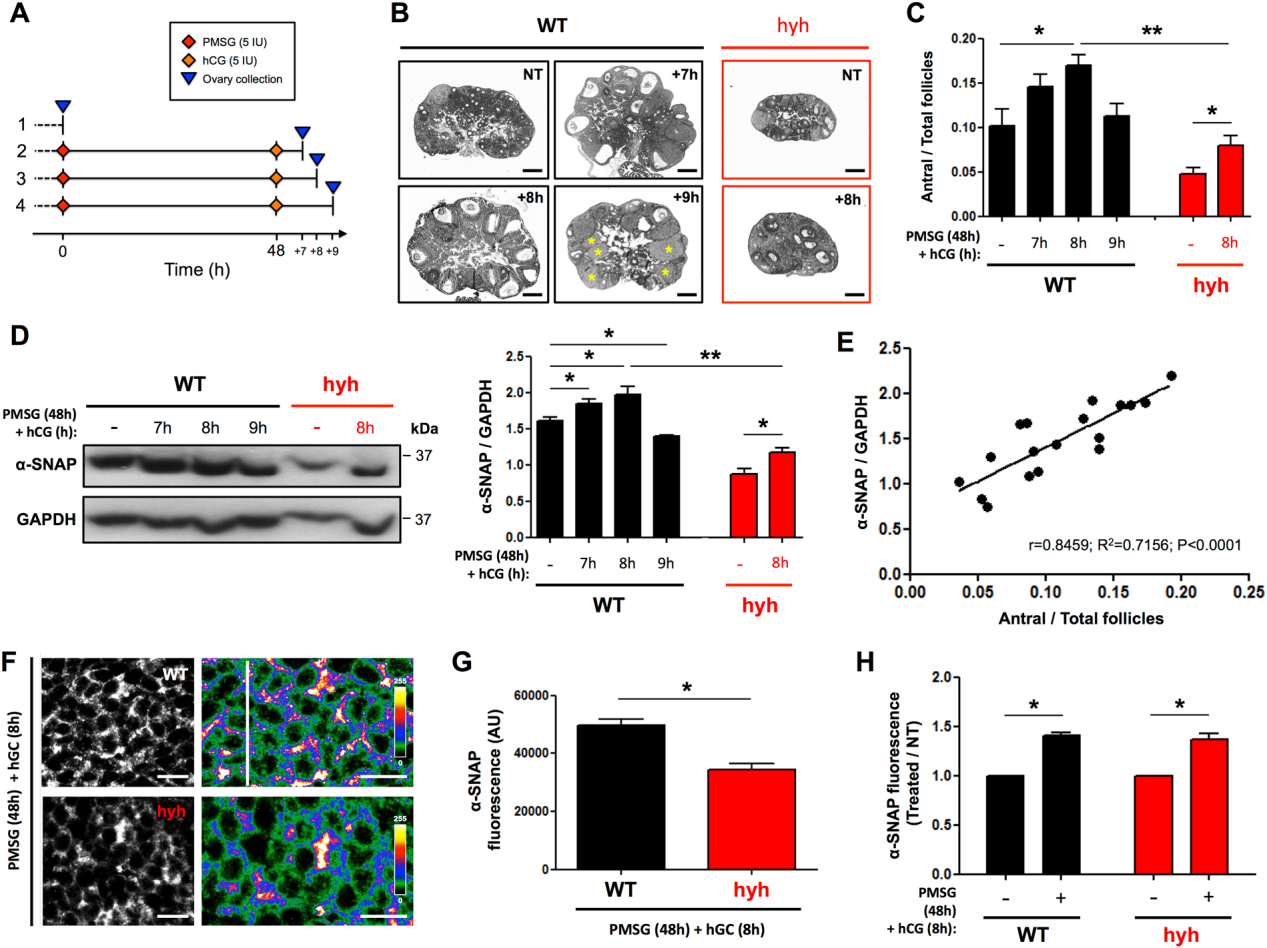
Expression of α-SNAP in ovarian tissue and granulosa cells (GCs) after gonadotropin stimulation. (**A,B**). Scheme of the protocol used for gonadotropin stimulation and ovarian tissue collection (**A**), and representative images of hematoxylin-eosin staining of ovary sections (**B**). Wild type (WT) females were divided into four groups: 1, control non-treated females (NT); 2–4, females received gonadotropin stimulation (one i.p. injection of PMSG (5 IU) + one i.p. injection of hCG (5 IU) 48 h after PMSG injection) and ovaries were collected 7 h (+7; group 2), 8 h (+8; group 3) and 9 h (+9; group 4) after hCG injection (see Material and Methods for details). Mutant females were included in protocol 1 (NT) and 3 (+8). Note the presence of abundant antral follicles in +7 and +8 WT ovaries; on the other hand, several corpora lutea are observed in +9 ovary (yellow asterisks). Scale bars, 200 μm. (**C**) Morphometric analysis of antral follicles in WT (black bars) and hyh (red bars) ovaries from control or non-treated females (−) and after PMSG + hCG treatment (7 h, 8 h, 9 h). (**D**) Western blot analysis of α-SNAP in ovary protein extracts obtained from WT and hyh non-treated females (−), and after PMSG + hCG treatment (7 h, 8 h, 9 h). GAPDH was used as loading control. Blots are representative of 3 independent experiments. Bars represent densitometric analysis of Western blots (mean ± SEM, n = 3). (**E**) Correlation (linear regression) analysis of normalized α-SNAP protein levels and the relative number of antral follicles. A strong positive linear correlation (r = 0.8459) and coefficient of determination (R^2^ = 0.7156) is observed (p < 0.0001). (**F**) α-SNAP immunolabeling in P60 WT and mutant hyh ovarian follicles 8 h after gonadotropin stimulation (PMSG + hCG). GC region in ovarian follicles are magnified and pseudocolored using the lookup table shown at the right of the figures to highlight the differences in fluorescence intensity between WT and mutant hyh samples. Scale bars, 10 μm. (**G**,**H**) Quantification of α-SNAP immunofluorescence intensity in WT (black bar) and hyh (red bar) GCs. Bars represent mean ± SEM of 4 independent experiments. (**H**) Relative α-SNAP immunofluorescence intensity in GCs from PMSG + hCG treated females compared with non-treated (control) females. Note that the increase in WT (black bars) is similar to that of hyh (red bars) GCs. *p < 0.05; **p < 0.01 (**C**,**D**, ANOVA with Tukey’s post hoc test; **G**,**H**, Student’s t-test).

The higher levels of α-SNAP protein in postpubertal (P60) ovaries/GCs suggested that ovarian α-SNAP expression might be associated with the continuous growth and maturation of ovarian follicles observed from puberty onwards. To elucidate whether this phenomenon was linked to the increased number of GCs in growing follicles, we decided to study α-SNAP expression in WT ovaries after gonadotropin stimulation (superovulation treatment) (Fig. 2A; see Material and Methods and Supplementary Information). Morphometric analysis of antral follicles (as a measure of the largest follicle population with the highest number of GCs) showed that at 7 h post-stimulation the relative number of antral follicles was increasing and at 8 h post-stimulation was significantly higher compared to controls (no treatment) (Fig. 2B and C). Similarly, Western blot analysis showed that α-SNAP protein increased significantly at 7 h and 8 h post-stimulation compared with controls (Fig. 2D). Interestingly, at 9 h post-stimulation, the relative number of antral follicles was not different from that observed in non-stimulated (control) ovaries (Fig. 2C), but the levels of α-SNAP protein were lower than those found in control ovaries (Fig. 2D). Remarkably, at this time point (9 h post-stimulation) the presence of corpora lutea was much more evident in stimulated ovaries than in non-stimulated ones (yellow asterisks in Fig. 2B), suggesting that massive ovulation starts at a time point between 8 h and 9 h post-stimulation. Considering these results, hyh mutant ovaries were studied only 8 h after hCG injection. The relative number of antral follicles and the levels of α-SNAP protein in hyh ovaries after gonadotropin stimulation were significantly lower than those in WT ovaries (Fig. 2B–D); however, both parameters were significantly increased when compared with non-stimulated hyh females (Fig. 2B–D), indicating a response to gonadotropin stimuli. Correlation analysis showed that there is a strong positive correlation between the relative number of antral follicles and the level of ovarian α-SNAP protein (Pearson’s correlation coefficient (r) = 0.8459; Fig. 2E). Interestingly, the coefficient of determination (R^2^ = 0.7156) suggests that ~72% of the total variation in ovarian α-SNAP protein levels can be explained by differences in the relative number of antral follicles and, consequently, the relative number of GCs. Thus, to assess whether increased ovarian α-SNAP protein levels after gonadotropin stimulation also implies an overexpression of α-SNAP in GCs, we analyzed gonadotropin-treated ovaries by indirect immunofluorescence. Despiteα-SNAP immunofluorescence intensity was lower in mutant GCs compared to WT GCs after gonadotropin treatment (Fig. 2F,G), we found that treatment increased α-SNAP fluorescence intensity in both WT and hyh mutant GCs compared with non-treated conditions (Fig. 2H; compare Fig. 2G with Fig. 1G). To confirm these results, purified GCs and ovarian remnant tissue obtained from P60 WT females subjected to superovulation treatment (8 h after hCG injection) were analyzed by Western blot. Interestingly, α-SNAP levels were increased in GCs after gonadotropin treatment(see Supplementary Fig. S6), suggesting that α-SNAP expression in GCs is hormonally regulated.

### Consequences of α-SNAP function deficiency (hyh mutation) in ovarian folliculogenesis and follicular atresia

**Figure 3:**
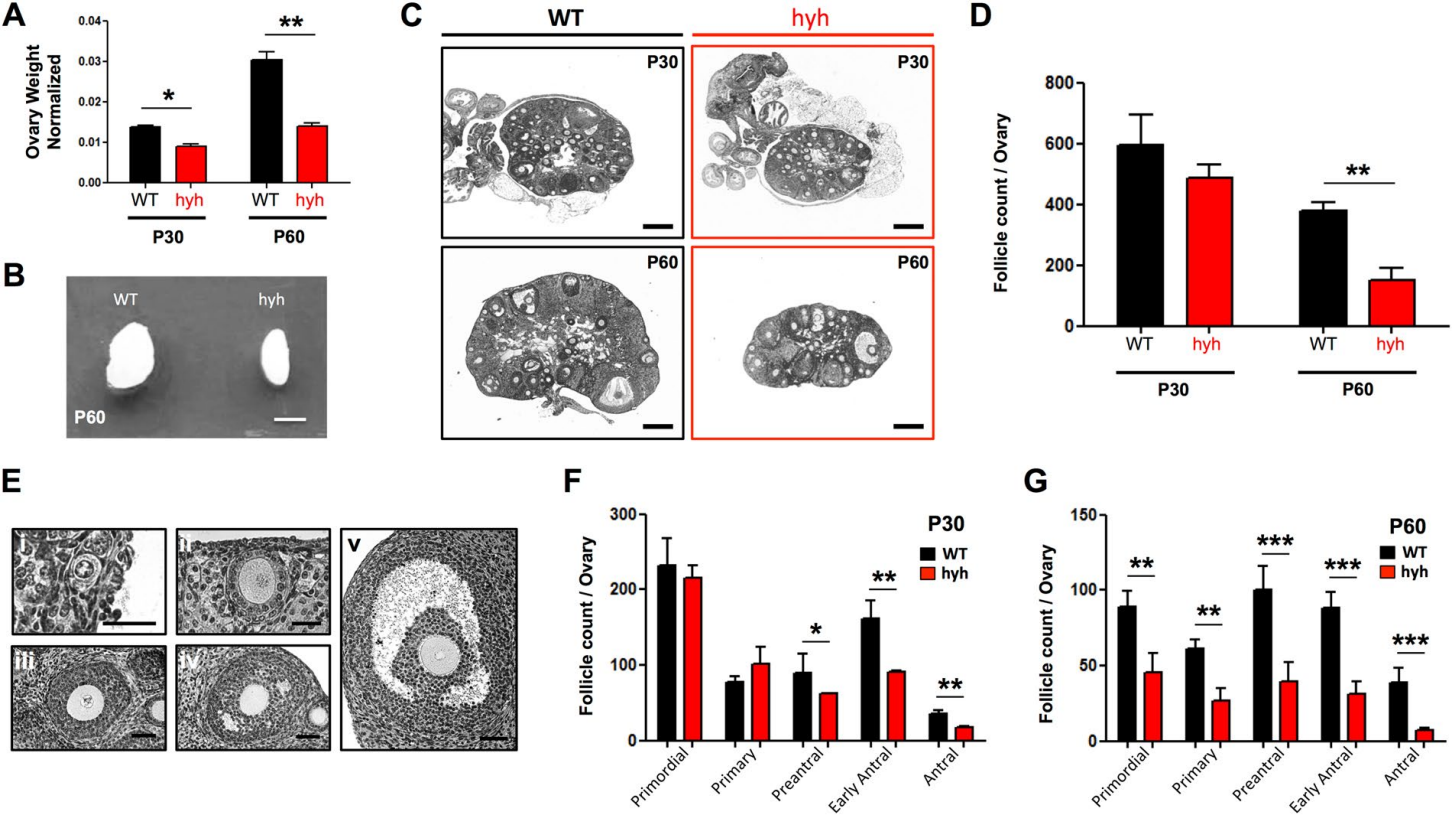
Ovarian phenotype and ovulation capacity of α-SNAP mutant (hyh) females. (**A**) Ovary relative to body weight in WT (black bars) and hyh mutant (red bars) P30 and P60 females. (**B**) Representative image of P60 WT and hyh ovaries. Scale bar, 2mm. (**C**) Representative histological sections of WT and hyh mutant ovaries at P30 and P60. Scale bars, 200 μm. (**D**) Number of follicles per ovary in P30 and P60 WT and hyh females. (**E**) Representative images of ovarian follicles at different developmental stages: primordial (i), primary (ii), preantral (iii), early antral (iv), and antral (v) follicles. Scale bars, 25 μm (i–ii) and 50 μm (iii–v). (**F**,**G**) The number of follicles in each stage per ovary was analyzed in P30 (**F**) and P60 (**G**) WT and mutant hyh ovarian histological serial sections. Bars represent mean ± SEM of 4 independent experiments. *p < 0.05; **p < 0.01; ***p < 0.001 (Student’s t-test).

Gonadotropin-modulated expression of α-SNAP in GCs suggests that α-SNAP may play a role in the folliculogenesis process. To test this hypothesis, we assessed whether hyh mutation of α-SNAP was associated with defects or changes in ovarian follicle development. To this end, we examined the ovarian phenotype of hyh mutant females and performed morphometric analyses of ovarian follicles at peripubertal (P30) and postpubertal (P60) stages. Considering that hyh mice are smaller than WT mice^48^, age-matched WT and hyh mutant females and their isolated ovaries were weighed (see Supplementary Fig. S7). When ovary weight was normalized to body weight we observed that P30 hyh ovaries were in average 33.3% smaller than WT ones, and this phenomenon was more evident in P60 hyh ovaries (52.5% smaller than WT ones) (Fig. 3A–C). In agreement with these results, follicle counts in ovarian serial sections showed that the total number of follicles was dramatically reduced in P60 hyh ovaries compared to WTs (Fig. 3D). To obtain detailed information about follicle development and to determine whether a particular stage of folliculogenesis is selectively affected in hyh mice, we classified ovarian follicles in primordial, primary, preantral, early antral and antral stages (Fig. 3E) and quantified the number of follicles in histological serial sections. Albeit we did not find differences between WT and hyh in the total number of follicles in P30 ovaries (Fig. 3D), we found a significant reduction in preantral, early antral and antral follicles (Fig. 3F). This fact was also evident when the relative number of follicles between WT and hyh females was analyzed (see Supplementary Fig. S8). These results are consistent with the relative distribution of follicles in each genotype. Hence, P30 WT ovaries showed that 52.2% of the follicles were primordial/primary follicles, and 33.6% were early antral/antral follicles. On the other hand, in P30 hyh ovaries, 64.8% of the follicles were primordial/primary follicles and only 22.4% were early antral/antral follicles (Supplementary Fig. S8). In P60 ovaries, we found a dramatic reduction in the relative number of follicles at all stages (Fig. 3G). In addition, when the relative decrease in each stage was compared with the relative reduction in the total number of follicles, we found that primordial and primary follicles were less affected than early antral/antral follicles (see Supplementary Fig. S8). Thus, the analysis of the relative distribution of P60 WT follicles showed that primordial and primary follicles represented a 39.6% of the total number of follicles, while early antral/antral follicles represented the 33.5% of the total. Conversely, in P60 hyh females, 48.6% of the total follicles were primordial and primary follicles, and 25.6% of the follicles were early antral/antral follicles (Supplementary Fig. S8). These results suggest that α-SNAP is relevant in folliculogenesis and a deficiency in α-SNAP function leads to (i) defects in the preservation of the number of follicles at postpubertal stages and (ii) a dramatic reduction in the number of follicles at the later stages of follicle development.

**Figure 4:**
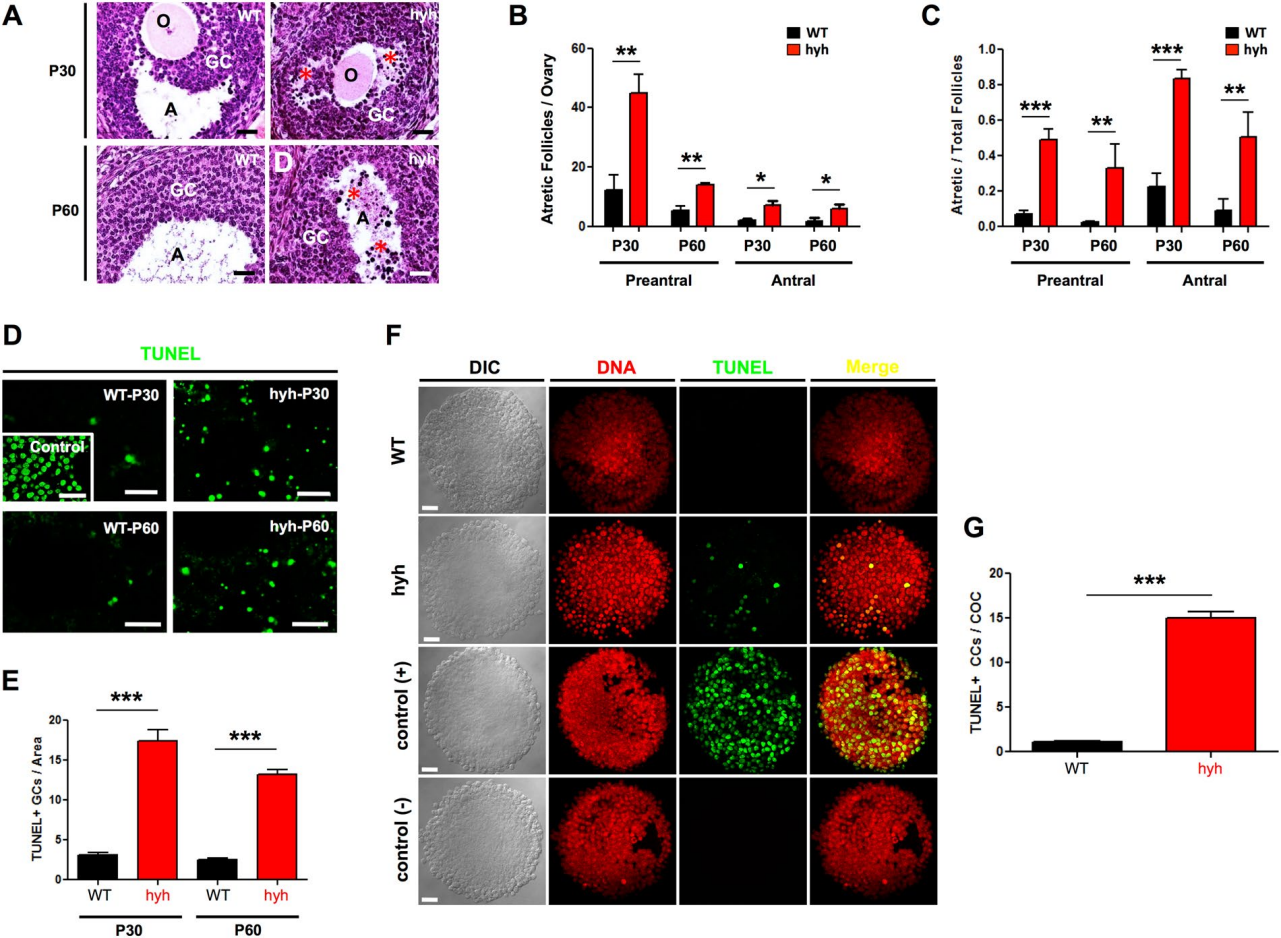
Follicular atresia and apoptosis of granulosa cells (GCs) in α-SNAP mutant (hyh) ovaries. (**A**) Sections of ovaries obtained from P30 and P60 WT and hyh mutant mice stained with hematoxylin-eosin. Magnifications of parts of the ovaries showing representative follicles. Atretic follicles characterized by the presence of condensed (pyknotic) nuclei (red asterisks) at the mural GC layers and in the follicular antrum (**A**) were abundant in hyh mutant ovaries. O, oocyte. Scale bars, 50 μm. (**B**) Number of preantral and antral atretic follicles per ovary in P30 and P60 WT and hyh females. Bars represent mean ± SEM of 4 independent experiments. (**C**) Quantification of the relative number of atretic follicles among preantral and antral follicles in WT and hyh mutant mice at P30 and P60. Follicles were scored as atretic if they demonstrate at least 5% pyknotic nuclei. Bars represent mean ± SEM of 4 independent experiments. (**D**) Representative images of TUNEL assays in histological sections of WT and hyh mutant ovaries at P30 and P60. For positive controls (control), histological sections were incubated with recombinant DNase I prior to labeling procedures. Scale bars, 50 μm. (**E**) Quantification of TUNEL + granulosa cells and bodies per area. Bars represent mean ± SEM of 3 independent experiments. (**F**) Representative images of TUNEL assays in COCs isolated from P60 WT and hyh ovaries 48 h after PMSG (10 IU) treatment. Propidium iodide was used for DNA detection (DNA). DIC, differential interference contrast microscopy. Scale bars, 20 μm. (**G**) Quantification of TUNEL + cumulus cells (CCs) per cumulus-oocyte-complex (COC). Twenty-nine WT and 33 hyh COCs obtained from 3 WT and 3 hyh females were analyzed. Bars represent mean ± SEM. ***p < 0.001 (Student’s t-test).

Interestingly, the histological analysis allowed not only identifying differences in the number of follicles but also to recognize that several follicles of hyh mutant ovaries were atretic^52,53^. Actually, abundant GCs showed cell shrinkage and pyknotic nuclei within the mural layers and in the antrum (Fig. 4A). Quantification of preantral and antral atretic follicles at P30 and P60 showed a dramatic increase of atresia in hyh mutant ovaries at both stages (Fig. 4B). Thus, the incidence of atresia (atretic/total follicles) in WT preantral follicles was 0.07 ± 0.02 (P30) and 0.03 ± 0.006 (P60), while in hyh mutant preantral follicles it was 0.49 ± 0.06 (P30) and 0.33 ± 0.14 (P60), representing an average of 7-fold (P30) and 10-fold (P60) increase in atretic preantral follicles in mutant ovaries compared with WT ones (Fig. 4C). On the other hand, the number of atretic/total follicles was 0.23 ± 0.07 and 0.09 ± 0.06 in WT P30 and P60, respectively; and 0.83 ± 0.05 (P30) and 0.51 ± 0.14 (P60) in mutant ovaries (Fig. 4C), which means a 3.6 and 5.7-fold increase, respectively.

To further support these results, the occurrence of apoptosis was analyzed in ovarian sections of P30 and P60 mutant and WT mice by terminal deoxynucleotidyl transferase-mediated nick end labeling (TUNEL) technique (Fig. 4D). In the WT ovaries of P30 and P60 some apoptotic nuclei were detected but most of the follicular tissue did not show signs of apoptosis. In contrast, in ovaries of P30 and P60 mutant animals, tissue sections stained for TUNEL showed intensive labeling of fragmented DNA in GC layers and in the antrum of the large follicles, which suggests that the apoptotic cells released their content into the follicular antrum. The number of TUNEL + cells/area was 5.6 and 5.3-fold higher in hyh mutant follicles compared with WT follicles (Fig. 4E). To further analyze the increased rate of apoptosis in hyh mutant GCs compared with controls, we decided to analyze the incidence of apoptosis in cumulus GCs using isolated cumulus-oocyte complexes (COCs) obtained 48 h after gonadotropin (PMSG 10 IU) treatment. Interestingly, cumulus GCs cells from hyh mutants showed a 14-fold higher incidence of apoptosis than that observed in WT cells (Fig. 4E). Hence, these results indicate that α-SNAP plays a key role in GCs survival and consequently in the balance between follicular development and follicular atresia.

### α-SNAP-mutant females present a reduced ovulation rate

**Figure 5:**
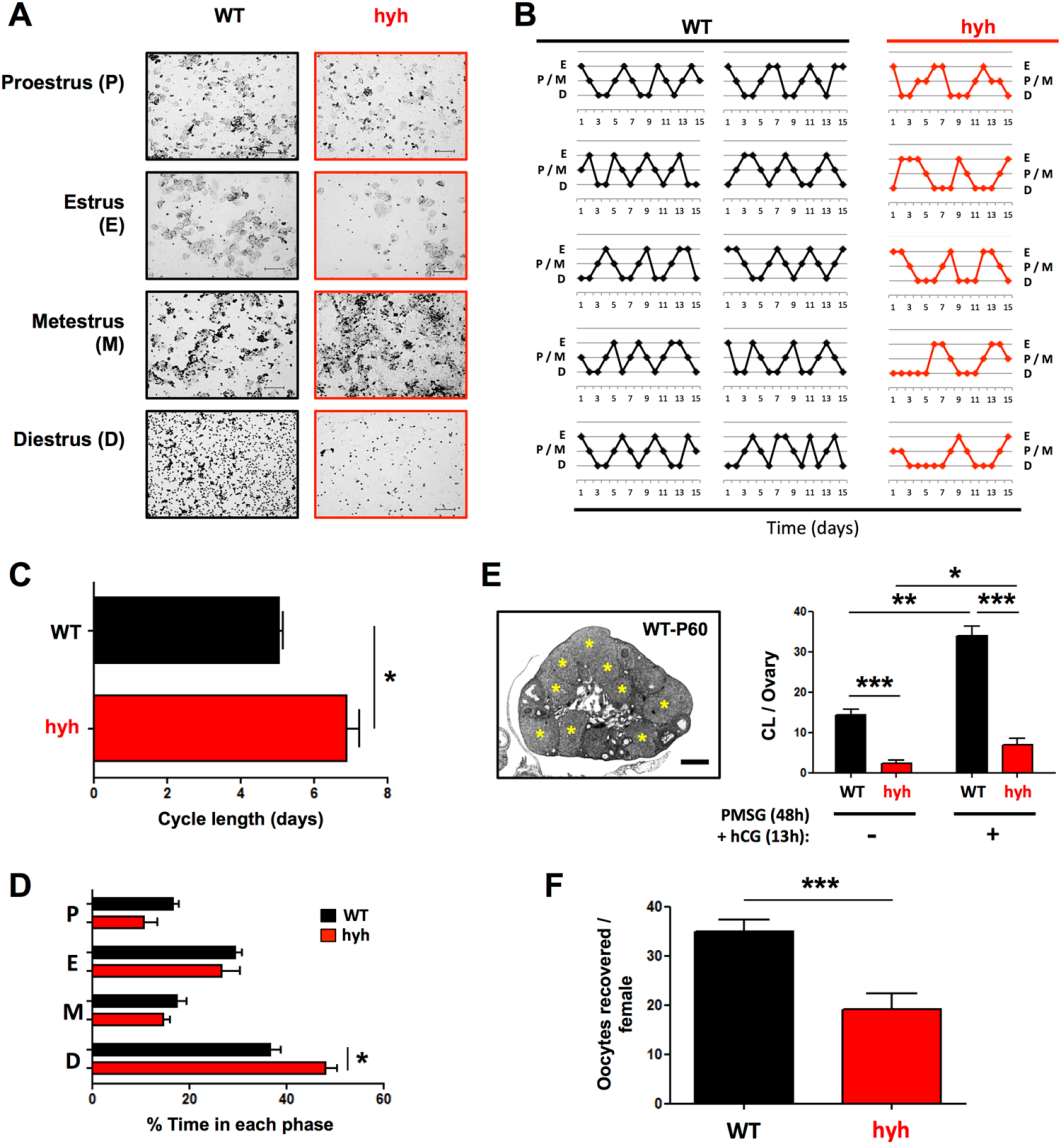
Estrous cycle and ovulation rate of α-SNAP mutant (hyh) females. (**A**) Estrous cycle phases in postpubertal WT and mutant hyh females. Representative images of the cytology of vaginal secretion. According to the relative abundance of nucleated epithelial cells, cornified epithelia cells and leucocytes, the cycle was divided in Proestrus (P), Estrus (E), Metestrus (M) and Diestrus (D). Note the abundance of cornified epithelial cells in E and leukocytes in D. (**B–D**) Daily analysis of vaginal secretion cytology during a 15 days period (n = 10 WT and 5 hyh). (**B**) Estrous cycles in hyh mutant females (red lines) are irregular compared with the cycles of WT females (black lines). (**C,D**) The average length of the estrous cycle was larger in hyh mutant females (**C**), and it was mainly due to a prolonged diestrus phase (**D**). Bars represent mean ± SEM (n = 10 WT and 5 hyh). (**E**) Quantification of corpora lutea (CL) in WT (black bars) and hyh (red bars) ovaries from non-treated (control) females (−) and 13 h after PMSG (48 h) + hCG treatment. A representative image of P60 WT ovary 13 h after gonadotropin stimulation is shown. Note the presence of abundant corpora lutea (yellow asterisks). Scale bar, 400 μm. Bars represent mean ± SEM of 4 independent experiments. (**F**) Quantification of MII oocytes collected from the oviductal ampulla of WT (black bars) and mutant hyh (red bars) females, 13 h after gonadotropin (PMSG × 48 h + hCG) stimulation. Bars represent mean ± SEM (n = 31 WT and 19 hyh). *p < 0.05; **p < 0.01; ***p < 0.001(Student’s t-test).

As hyh females showed an increased incidence of follicular atresia, we analyzed the impact or consequences of this phenomenon on ovulation capacity. To analyze ovulation rate, we first examined estrous cyclicity. Interestingly, all sexually mature mutant females analyzed exhibited vaginal cytology consistent with active estrous cycles (Fig. 5A,B). Sequential analysis of vaginal cytology of WT and age-matched hyh females throughout a continuous period of 15 days (60 to 75 days old) showed that (i) cycles of hyh mutant females were more irregular than those of WT females (Fig. 5B), and (ii) the average cycle length was larger in hyh females (Fig. 5C). Hyh females presented an average of 1.6 cycles in a 15-days period, while WT females showed an average of 2.7 cycles in the same period (see Supplementary Fig. S9). Interestingly, when the time spent in each estrous phase was analyzed, we found that hyh females spent the same relative time in estrus than WT females but they spent extended periods of time in diestrus phase (Fig. 5D). Thus, the extended estrous cycles observed in hyh females appear to be due mainly to an over-representation of diestrus phase.

In order to quantify the ovulation rate of hyh mutant females, the ovaries of age-matched WT and hyh mice in metestrus phase were processed for histology and the number of corpora lutea (CL) was analyzed. In addition, CL were also quantified in gonadotropin-stimulated (13 h after PMSG/hCG treatment) mice. Consistent with previous findings, the number of CL was significantly reduced in hyh females, independently of the hCG treatment (Fig. 5E). To confirm that the reduction in the number of CL is indeed associated to a decline in ovulation rate, we quantify the number of MII oocytes collected from the oviductal ampulla 13 h after PMSG/hCG treatment. As expected, the number of MII oocytes recovered per female was also significantly reduced in hyh mice compared with WT controls (Fig. 5F).

### α-SNAP-mutant females show a dramatic decline in reproductive efficiency

**Figure 6:**
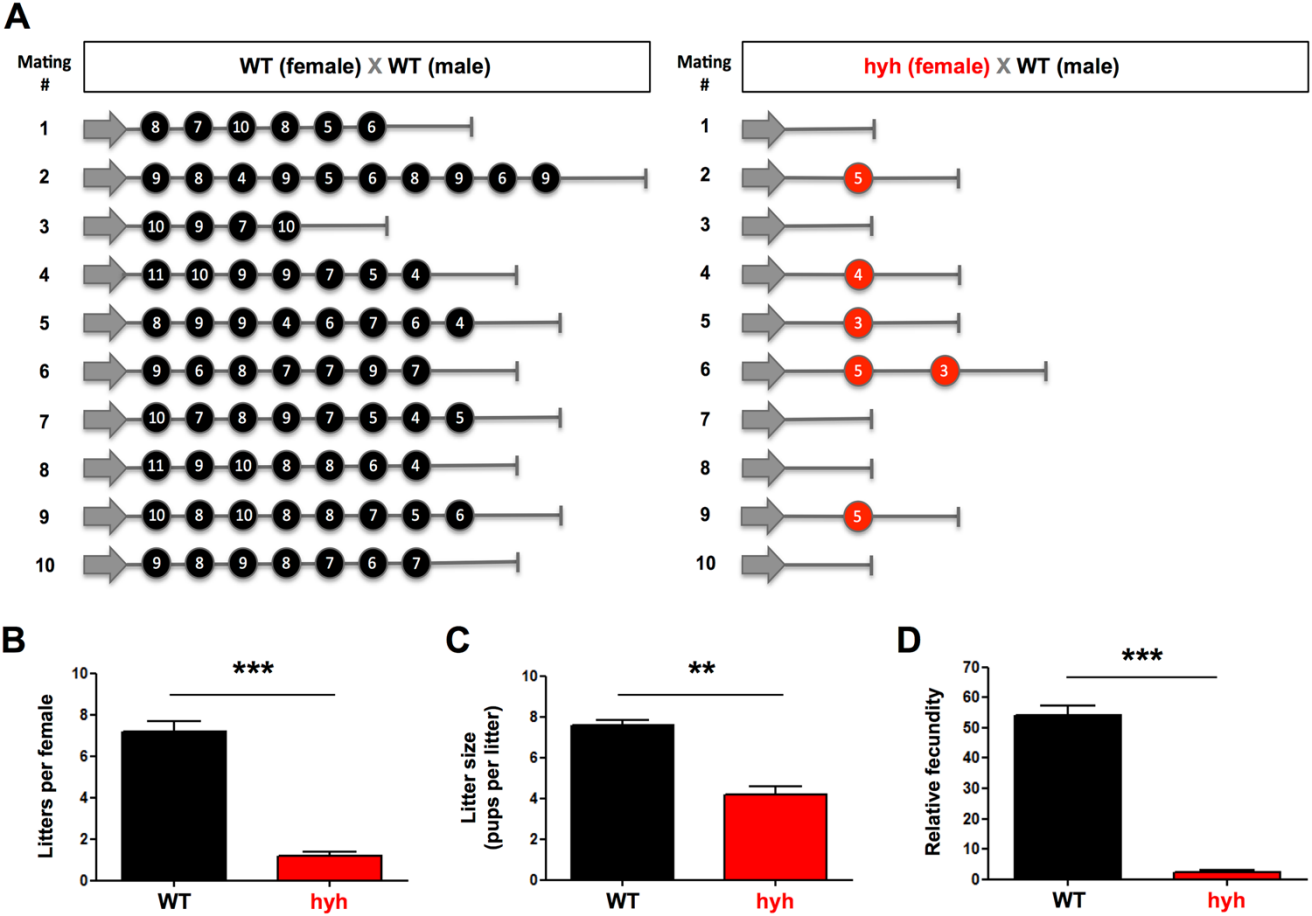
Reproductive performance of α-SNAP mutant (hyh) females. (**A**) Schematic representation of the reproductive profile of WT (n = 10) and hyh mutant (n = 10) females. Each circle represents a litter and the number inside the circle represents the litter size (pups born in that litter). (**B–D**) Quantification of the number of litters per female (**B**), litter size (**C**) and relative fecundity (**D**). Relative fecundity was obtained as: (litter size) × (number of litters) × (productive matings/100); the obtained value is a measure of the overall fecundity according to the Handbook of Genetically Standardized JAX Mice. Bars represent mean ± SEM (n = 10 WT and 10 hyh). **p < 0.01; ***p < 0.001 (Student’s t-test).

We then analyzed the reproductive performance of female hyh mutant mice. For this purpose, female hyh mutants and their corresponding female WT littermates were mated with WT male mice of proven fertility, and each mating was individually registered. Figure 6A graphically represents the reproductive performance observed in both groups. All WT mating pairs were productive (i.e., at least one offspring was born) and 8 out of 10 produce 7 or more litters. On the other hand, only 50% of hyh mating pairs were productive with four pairs having only one litter and one pair two litters (Fig. 6A). Remarkably, a vaginal plug was frequently observed in most of the females that had no productive matings, suggesting that they were receptive to copulate but were not able to complete pregnancy and/or gestation. The number of litters per female was noticeably reduced in hyh female mice (Fig. 6B). Additionally, the number of pups per litter (litter size) was in average 7,6 in WT mating pairs and 4,2 in hyh mating pairs (only productive pairs were considered) (Fig. 6C). Finally, we calculate the relative overall fecundity of WT and hyh females according to previous studies^50^. Hyh females showed a dramatic reduction of this index from 54.1 in WT females to 2.5 in hyh females (Fig. 6D). These results strongly suggest that α-SNAP function is necessary for female fertility and that hyh (M105I) mutation provokes severe deficiencies in female reproductive performance.

## Discussion

The present study shows that α-SNAP is particularly enriched in ovarian GCs. In addition, the hyh (M105I) mutation of α-SNAP yields a discernible ovarian phenotype, characterized by increased apoptosis of GCs, a premature decline in ovarian follicular reserve and defects in ovulation rate. Consequently, homozygous hyh mutant females are severely subfertile compared with WT females.

Since α-SNAP is a ubiquitously expressed multifunctional protein, is likely that hyh mutant females display a multifactorial reproductive phenotype and that several mechanisms might be involved in its pathogenesis. Thus, we cannot exclude the possibility that the ovarian phenotype of hyh mutant mice may partially result from changes in the function of the hypothalamic-pituitary-ovary (HPO) axis and an exhaustive study addressing the function of hypothalamic neurons, the dynamic release of pituitary hormones and the influence of other neuroendocrine factors would help to better understand the reproductive phenotype complexity of α-SNAP mutant mice. On the other hand, it is known that the release of neurotransmitters and neuropeptides during neuronal signaling is triggered by Ca2+ ions and executed by the NSF/SNAP/SNARE protein machinery. Interestingly, in neurons, the SNAP-dependent SNARE complex disassembly (activation) is mediated not only by α-SNAP but also by β-SNAP. In fact, the expression of β-SNAP is restricted to the nervous tissue and is only detectable postnatally^54,55^. Thus, in postnatal mouse brain, α- and β-SNAP are co-expressed and both proteins are able to support synaptic transmission. It has been shown that, despite the reduction in α-SNAP function, neurons obtained from hyh mutant mice show no defects in basic parameters of synaptic transmission and plasticity^36^. Similarly, neurons obtained from β-SNAP KO mice preserve synaptic parameters. Instead, neurons obtained from double-mutant (α-SNAP.hyh/ β-SNAP.KO) mice present impairments in synaptic function, indicating that both proteins present a functional equivalence and both support neuronal firing (neurotransmitter release)^36^. Hence, in adult hyh mice it can be expected that neuronal synaptic function be preserved at a certain level by β-SNAP function.

In line with this view, the evaluation of estrous cycle in hyh mutants showed that all females analyzed presented cyclicity, indicating that the HPO axis is at least partially functional. Furthermore, 50% of hyh females are able to have productive matings.

The hyh (M105I) mutation resides in a region of α-SNAP that does not interact with the SNARE complex^56^. *In vitro* experiments have shown that the protein harboring the M105I mutation can bind to and disassemble SNARE complexes in a way similar to the wild type protein^42^. These observations, together with the fact that the hyh mouse presents a diminished amount of α-SNAP protein (hypomorphism) in several tissues, have lead to the assumption that the hyh phenotype is due to hypomorphism and not to a malfunction of the mutated protein^42,43^. However, a functional defect of the mutated protein has not been ruled out. Moreover, studies performed in hyh sperm (cellular context) indicate that α-SNAP (M105I) protein has an intrinsic malfunction^50^. These apparently contradictory results suggest that the M105I mutation may interfere (loss of function) or promote (gain of function) the interaction of α-SNAP with cellular components not present in *in vitro* assays, such as membrane lipids^57^ or non-SNARE interacting proteins^39^.

This is, to the best of our knowledge, the first study where α-SNAP protein is comprehensively examined in ovarian tissue, showing that α-SNAP protein is expressed in mouse ovarian GCs. Other studies have documented the expression of α-SNAP mRNA in isolated rat GCs obtained from prepubertal animals, with contradictory results^58,59^. DNA microarray analysis performed by Jo and coworkers indicate that α-SNAP mRNA is highly expressed in isolated GCs^58^. On the other hand, Lin and coworkers were not able to detect α-SNAP mRNA by qPCR in isolated GCs^59^. Methodological differences between both experimental designs may explain such opposing results.

Our study shows that α-SNAP expression in ovarian tissue and GCs is particularly enriched in postpubertal stages. Similarly, exogenous gonadotropin stimulation increased α-SNAP protein levels, suggesting that α-SNAP expression is hormonally regulated. Interestingly, other proteins associated with the SNARE-mediated membrane fusion machinery, such as sinaptosomal-associated protein of 25 kDa (SNAP-25) and synaptotagmin VII, have been previously described in ovarian tissue and GCs^60,61^. Furthermore, the expression of SNAP-25 by GCs is hormonally regulated^27^. Gonadotropin (FSH) stimulation increases SNAP-25 mRNA and protein levels in GCs via cAMP/PKA pathway^61,62^. Together, these results strongly suggest that α-SNAP function (i) may play a vital role in gonadotropin-induced pathways during follicular growth, differentiation, and survival, and (ii) may be coupled to SNAP-25 function. It has been recently demonstrated that α-SNAP and SNAP-25 mediate cholesterol movement to mitochondria and support steroidogenesis in different cell types^59^. Additionally, GCs secrete, via exocytosis, a wide variety of factors such as activin, anti-Müllerian hormone (AMH), bone morphogenetic proteins (BMPs) and fibroblast growth factors (FGFs), that modulate folliculogenesis (reviewed in^63^). It has been demonstrated that secretion of cytokines and chemokines by GCs is SNAP-25-dependent^27^. Taken together, these studies indicate that, in GCs, the α-SNAP/SNARE machinery is necessary to mediate both the trafficking/exocytosis processes, and the increased synthesis and secretion of steroids after gonadotropin stimulation. In line with this view, hyh females showed prolonged interestrous intervals mainly due to extended periods of diestrus. As low circulating estrogen is associated to prolonged diestrus^64^, these results highlight the putative role of α-SNAP in the steroidogenic process in GCs.

Even though gonadotropins increase α-SNAP levels in the ovary and GCs, the hypomorphism observed in hyh GCs and ovarian tissue does not seem to be a direct consequence of a putative reduction in gonadotropin stimuli. Besides, and as it has been previously proposed, it seems that α-SNAP hypomorphism in hyh tissues is a consequence of a reduction in the stability of mutant α-SNAP mRNA and/or protein^42,43^. In fact, in this and in other studies it has been demonstrated that α-SNAP levels are reduced in several tissues that do not express FSH receptor^42,43,45,50^. Exogenous gonadotropin treatment is able to increase α-SNAP levels in WT and hyh mutant GCs, suggesting that signaling components downstream gonadotropin receptor are preserved in hyh mutant GCs. However, exogenous gonadotropin treatment is not able to “rescue” neither α-SNAP levels nor ovarian phenotype in hyh mice. Thus, intra-ovarian (cell-autonomous?) defects are likely to be key determinants of the hyh phenotype.

Our present results reveal for the first time a mouse ovarian phenotype compatible with premature ovarian insufficiency or failure (POI or POF) in mice associated with a defective function in α-SNAP. POI describes an accelerated decline of ovarian function resulting in an earlier than average menopause^65^. It is believed that 1% of women under 40 years and 0.1% under the age of 30 years will develop POI^8^. In most cases, the etiology is unexplained and a strong genetic component is suspected^66,67^. Some candidate genes associated to POI such as growth differentiation factor-9 (GDF-9) and bone morphogenetic protein-15 (BMP-15) appears to affect ovarian reserve in a gene dosage-dependent fashion. In fact, results obtained in spontaneously mutant sheep indicate that only homozygous carriers of mutations in those genes have impaired fertility, whereas heterozygous display increased fertility^68^. In mice, the haploinsufficiency of kisspeptin receptor (Kiss1r) induces a state of POF^69^. We have observed that heterozygous hyh females present an ovarian and reproductive phenotype indistinguishable from that of WT females (see Supplementary Fig. S10;^50^), hence the consequences of M105I (hyh) mutation appear to be gene dosage-dependent and only homozygous mutant hyh females show an apparent ovarian and reproductive phenotype.

One of the pathogenic mechanisms associated with POI is accelerated follicular atresia^70^. In addition, several studies indicate that apoptosis of GCs triggers or initiates atresia of ovarian follicles in mammals^71–76^. Our results provide strong evidence that the ovarian phenotype observed in hyh mutant mice is due, at least in part, to an increased rate of apoptosis in GCs and follicular atresia. As a multifunctional protein, α-SNAP appears to coordinate intracellular membrane trafficking/fusion with other relevant cellular processes such as cell adhesion, autophagy, AMPK activity, and apoptosis^37–41^. In fact, it has been described that α-SNAP has anti-apoptotic functions by modulating Bcl-2 expression^41^ and by interacting with BH3-only proteins such as BNIP1^77^. Furthermore, α-SNAP can modulate cell survival by acting as a negative regulator of autophagy^40^. It has been proposed that a functional interplay between autophagy and apoptosis in GCs play a significant role in the balance between follicular development and atresia^14,78,79^. These studies suggest that autophagy is directly involved in follicular atresia and regulates apoptotic cell death of GCs during folliculogenesis. Interestingly, toxicant-induced increase of autophagy in GCs induces depletion (atresia) of ovarian follicles^80^, and this detrimental effect is produced by a deregulation (activation) of the AMPK pathway^81^. Attractively, *in vitro* studies have shown that α-SNAP can negatively control AMPK signaling by acting as a phosphatase^39^. It has also been proposed that α-SNAP regulates integrin processing/trafficking^38^ and the assembly of cadherin-dependent junctions^37^. Interestingly, cadherin-mediated adhesion prevents apoptosis of GCs and follicular atresia^18,82,83^, and laminin-integrin interaction enhances survival and proliferation of GCs^84^.This evidence points out that several α-SNAP-dependent cell survival mechanisms may be altered in the GCs of hyh mutants and, consequently, further studies are needed to determine the precise mechanisms linking α-SNAP dysfunction with increased apoptosis of GCs. However, our results lend support to the notion that the function of α-SNAP is required for an adequate maintenance of follicular integrity particularly from puberty onwards. Furthermore, the function of α-SNAP appears to be relevant in later stages of follicular development, specifically in the transition from preantral to antral follicles. It is known that this transition (i) represents a change from gonadotropin-independent stages to gonadotropin-responsive and gonadotropin-dependent stages^72,85^, and (ii) is the phase where atresia rate is more noticeable^72^. As stated before, our results clearly show that gonadotropin stimulation increases the levels of α-SNAP protein in GCs and ovarian tissue. Thus, α-SNAP function may be relevant in gonadotropin-induced pathways involved in later stages of folliculogenesis such as regulating the balance between ovulation and atresia.

Finally, the dramatic decline in fertility observed in α-SNAP-mutant females suggest that, in addition to the impairment in folliculogenesis/ovulation process, other mechanisms including oocyte-related factors, a reduction in the number of cleaved embryos, implantation failure, early embryonic fetal loss, and changes in uterine environment may contribute to an additive detrimental effect of hyh mutation on female fertility. We have recently documented that α-SNAP is expressed in the cortical region of isolated mouse oocytes and mediates cortical granule exocytosis (CGE)^34^. CGE prevents polyspermy and, thus, guarantees the success of fertilization and embryo development since polyspermy is a lethal embryonic condition^86^. Preliminary experiments that have been undertaken in our labs to evaluate CGE in hyh oocytes suggest that hyh mutant oocytes undergo a defective cortical reaction (unpublished results). Consequently, a malfunction of α-SNAP in hyh oocytes may probably contribute or be an alternative explanation to the lowered fertility observed in hyh females.

Based on collective findings, we conclude that α-SNAP plays a critical role in the physiology of GCs, regulating the balance between folliculogenesis and follicular atresia. Hence, a reduction in its function (hyh mutation) causes increased incidence of apoptosis in GCs, early depletion of functional ovarian follicles, reduced ovulation rate, and female subfertility. Future research should focus on the cellular and molecular mechanisms by which α-SNAP preserve follicular development and female fertility. Such knowledge may have profound implications for understanding the regulation of ovarian follicle development and the pathogenesis of ovarian defects in human diseases.

## Methods

### Animals

Mice (B6C3Fe-a/a-Napa^hyh^/J) were obtained from The Jackson Laboratory (Bar Harbor, ME), where the hyh mutation arose spontaneously on the C57BL/10 J background and was then outcrossed onto a B6C3Fe-a/a (C57BL/6 J female X C3HeB/FeJ-a/a male) hybrid background^44^. Wild type (Napa^+/+^) and homozygous hyh mutant (Napa^hyh/hyh^) mice are designed here as ‘WT’ and ‘hyh’, respectively. All animals were genotyped by a PCR-based method described before^45^. Housing, handling, care and processing of the animals were carried out in strict accordance with the recommendations of the Guide for the Care and Use of Animals of the National Institutes of Health and the Institutional Animal Care and Use Committee of the Universidad Austral de Chile approved the experiments and protocols.

### Purified granulosa cells isolation and protein extraction

To isolate mouse granulosa cells (GCs), ovaries were removed from WT mice at postnatal day (P) 30, P60, and P60 subjected to gonadotropin (superovulation) treatment (see below; ovaries were collected 8 h after hCG injection), pooled and processed as previously described^87,88^ with slight modifications. The resultant GC and remnant tissue extracts were frozen at −80 °C until analyzed by SDS-PAGE and Western blotting.

### Light microscopy, morphometric analysis and indirect immunofluorescence

Ovaries were rapidly removed from WT and hyh mutant mice at P30 and P60, fixed in Bouin´s fixative solution for 48 h at room temperature (RT) and processed for hematoxylin and eosin staining and light microscopy studies. Morphometric analysis of ovarian follicles was performed as previously described^89,90^ with some modifications. Indirect immunofluorescence staining was performed on paraffin-embedded ovary sections and evaluated in a confocal laser microscope (Olympus Fluoview FV1000 microscope, at Universidad Austral de Chile). Images were processed and analyzed using the Image J software (NIH, Bethesda, MD).

### Protein extraction from tissue samples, production and purification of recombinant proteins, SDS-PAGE and immunoblotting

Proteins from WT and hyh tissues were extracted and analyzed by SDS-PAGE and Western blotting as described in^50^, with minor modifications. Generation of α-SNAP constructs and purification of recombinant proteins was performed as previously described^91,92^, with some modifications. Plasmid pcDNA3.1 encoding α-SNAP WT and plasmid pET28a encoding α-SNAP M105I (kindly provided by Dr. Phillys Hanson, Washington University, St. Louis, Missouri, USA) were used as templates.

### Gonadotropin (superovulation) treatment and oocyte collection

Gonadotropin stimulation of female mice was performed as previously described^50,93^.

### Determination of estrous cycle stage by vaginal cytology

The stage of the estrous cycle (diestrus, proestrus, estrus, or metestrus) was determined based on the relative presence of leukocytes (L), cornified epithelial (C), and nucleated epithelial cells (N) according to Felicio *et al*.^94^ and using the visual identification tool described by Byers *et al*.^95^.

### Data availability

The datasets generated during and/or analyzed during the current study are available from the corresponding author on reasonable request. Additional and detailed information regarding materials, experimental procedures and data analysis can be found in Supplementary Information.

## Electronic supplementary material

Supplementary Information
