# Publisher Correction: α-SNAP is expressed in mouse ovarian granulosa cells and plays a key role in folliculogenesis and female fertility

**DOI:** 10.1038/s41598-020-61863-w

**Published:** 2020-03-25

**Authors:** Alexis Arcos, Matilde de Paola, Diego Gianetti, Diego Acuña, Zahady D. Velásquez, María Paz Miró, Gabriela Toro, Bryan Hinrichsen, Rosa Iris Muñoz, Yimo Lin, Gonzalo A. Mardones, Pamela Ehrenfeld, Francisco J. Rivera, Marcela A. Michaut, Luis Federico Batiz

**Affiliations:** 1 Instituto de Anatomía, Histología y Patología, Facultad de Medicina, Universidad Austral de Chile, Valdivia, Chile; 2 Instituto de Histología y Embriología (IHEM), Universidad Nacional de Cuyo-CONICET, Mendoza, Argentina; 3 Instituto de Fisiología, Facultad de Medicina, Universidad Austral de Chile, Valdivia, Chile; 4 Center for Interdisciplinary Studies on the Nervous System (CISNe), Universidad Austral de Chile, Valdivia, Chile; 5 Institute of Molecular Regenerative Medicine, Paracelsus Medical University Salzburg, Salzburg, A-5020 Austria; 6 Spinal Cord Injury and Tissue Regeneration Center Salzburg, Paracelsus Medical University Salzburg, Salzburg, A-5020 Austria; 7 Facultad de Ciencias Exactas y Naturales, Universidad Nacional de Cuyo, Mendoza, Argentina; 8 Centro de Investigación Biomédica (CIB), Facultad de Medicina, Universidad de los Andes, Santiago, Chile; 9 Present Address: Department of Neurosurgery, Oregon Health and Science University, Portland, Oregon USA

Correction to: *Scientific Reports* 10.1038/s41598-017-12292-9, published online 18 September 2017

In the original version of this Article, the author Matilde de Paola was incorrectly indexed. This error has now been corrected.

