# Supplementary material for: α-SNAP is expressed in mouse ovarian granulosa cells and plays a key role in folliculogenesis and female fertility
Source: Sci Rep. 2017 Sep 18;7:11765. doi: 10.1038/s41598-017-12292-9 (PMC5603506; doi:10.1038/s41598-017-12292-9)
Supplement: Supplementary file 1 — Supplementary Information [file 41598_2017_12292_MOESM1_ESM.pdf]

Supplementary Material and Methods

α-SNAP is expressed in mouse ovarian granulosa cells and plays a key role in folliculogenesis and female fertility.

Alexis Arcos, Matilde de Paola, Diego Gianetti, Diego Acuña, Zahady D. Velásquez, María Paz Miró, Gabriela Toro, Bryan Hinrichsen, Rosa Iris Muñoz, Yimo Lin, Gonzalo A. Mardones, Pamela Ehrenfeld, Francisco J. Rivera, Marcela A. Michaut, Luis Federico Batiz

Animals

Housing, handling, care and processing of the animals were carried out in strict accordance with the recommendations of the Guide for the Care and Use of Animals of the National Institutes of Health and the Institutional Animal Care and Use Committee of the Universidad Austral de Chile approved the protocol. The mice were fed ad libitum with rodent food and maintained under a constant photoperiod of light/dark 12:12 h and room temperature of 25°C. Animals corresponding to the prepubertal period (postnatal day 7 and 14; P7 and P14), peripubertal period (P30), and postpubertal period (P60 and P120) were used in this study. According to the classification proposed previously that considers the clinical phenotype and the survival rate (48), and to discard effects provoked by a severe pathological (RP) phenotype, only female hyh mutant mice showing the mild (SP) phenotype were studied.

Purified granulosa cell isolation and protein extraction

Ovaries were subjected to puncture with a 26 gauge needle to liberate GCs into collection media (DME-F12, 1X penicillin-streptomycin, 0.3% BSA). Before puncturing ovaries were incubated in medium containing 0.5 M sucrose and 10 mM EGTA at 37 °C for 30 min, and were washed in fresh DMEM-F12 (83, 84). Resulting GC suspensions from both ovaries were pooled (mice were maintained as individual samples) and filtered through 40µm nylon filter to remove large tissue clumps and oocytes. The GC-depleted residual ovarian tissue (termed here as remnant tissue) was washed with

PBS and dounced in low salt protein extraction buffer. Fractions enriched in GCs were spun down (500 x g for 5 min), washed with PBS, spun down again, and resuspended in extraction buffer. Due to heterogeneity and complexity of the ovary, it is nearly impossible to isolate completely pure populations of specific ovarian cell types in rodents (52). Thus, there is likely to be a minor contamination of other cell types in our samples of GCs. Since it has been shown that N-cadherin (Ncad) is highly and preferentially expressed in mammalian GCs (18, 51), the densitometric ratio Ncad/H3 in Western blot studies was used as an index of GC purity. P60 "GC" index was established as the reference index (= 1) to calculate the purity index of the other fractions. Purity index were as follows (Mean ± SD): P60 "Re" = 0.04 ± 0.01; P30 "GC" = 0.98 ± 0.05; and P30 "Re" = 0.39 ± 0.04, showing that "GC" fractions were highly enriched in GCs compared with "Re" fractions, and that the degree of GC "purity" in the fractions obtained from P30 and P60 ovaries is comparable.

Sample preparation for Light microscopy

Ovaries from WT and hyh mutant mice at different developmental stages were fixed, dehydrated and embedded in paraffin (Paraplast; Sigma-Aldrich, St. Louis MO). Serial sections (6 µm thick) of the ovarian were obtained and mounted on silanized (3-aminopropyltriethoxysilane; Polysciences Inc., Warrington, PA) slides and used for routine hematoxylin and eosin staining. Sections were examined using a Zeiss Axioskop Microscope coupled to a digital camera (Axiocam, Zeiss).

Immunofluorescence and confocal microscopy

Sections were sequentially incubated in primary antibody for 18 h at room temperature and then in secondary antibody for 30 min at RT and in darkness. A mouse monoclonal anti-α-SNAP antibody (1:500; Exalpha Biologicals Inc., X1025) and a goat anti-mouse IgG labeled with Alexa488 (1:500; Life Technologies, A11001) were used as primary and secondary antibodies, respectively. All antibodies were diluted in Tris buffer, pH 7.8, containing 0.7% non-gelling seaweed gelatin lambda carrageenan and 0.5% Triton-X 100 (both from Sigma, St. Louis, MO). The omission of the incubation in the primary antibody was used as a control of the immunoreaction.

Sections were mounted in Vectashield (DAKO) and evaluated by confocal laser microscopy. Single channel gray images were subjected to pseudocolor (or indexed color) processing according to gray values (fluorescence intensity) using the Image J software (National Institutes of Health – NIH, Bethesda, MD). For α-SNAP immunofluorescence quantification, 3 sections per ovary were analyzed (n=4 animals per genotype). At least five follicles per section were pictured using 40X objective, and 2 to 4 non-overlapping regions of interest (ROI) of 1,500μm^2 in the GC layers were randomly defined in each follicle. Preantral, early antral and antral follicles were included in quantification analyses. The intensity of immunostaining was evaluated by measuring the mean grey value (Imaje J software), which is defined as the average of the pixel grayscale values contained in the ROI.

Protein extraction from tissue samples

Proteins from WT and hyh brain, cerebellum, heart, liver and ovary (at different postnatal stages) were extracted by homogenizing tissues in a modified RIPA buffer (50 mM Tris-HCl, pH 7.4, 150 mM NaCl, 1% Triton-X-100, 1% sodium deoxycholate, 0.1% SDS) with a protease inhibitor cocktail (P2714, Sigma Chemical Co.) plus 1 mM phenylmethanesulphonylfluoride (PMSF) using the same protocol described in (50). The homogenates were centrifuged at 6000 x g for 10 min at 4ºC to sediment unbroken cells and nuclei. Supernatants were stored at -80ºC until used.

SDS-PAGE and immunoblotting

For Western blotting, 2% (v/v) 2-mercaptoethanol was added to the samples. After boiling for 2 min, 30 μg of total protein were loaded on 10% polyacrylamide gels. The proteins were then transferred to polyvinylidene difluoride (PVDF) membranes (Millipore, Bedford, MA). Blots were incubated for 1–2 h at 37ºC with anti-α-SNAP (1:1000; Exalpha Biologicals Inc., X1025) diluted in PBS containing 0.05% Tween 20 and 1% BSA. Horseradish peroxidase conjugated goat anti-mouse-IgG was used as secondary antibody (0.25 mg/ml) with 30 min incubations at 37ºC. Excess antibodies were removed by washing 5 x 5 min in 1x PBS. Detection was accomplished using an enhanced chemiluminescence (ECL) kit (SuperSignal West Pico Chemiluminescent

Substrate, Pierce, Rockford IL) and imaging was obtained using the G:BOX XX6 chemiluminescence system and the Genesys Software (Syngene, Frederick MD). To confirm equal loading of proteins, blots were assayed with different loading controls: (i) anti-GAPDH antibody (Exalpha Biological Ic. X2414P) was used to compare different WT tissue homogenates, and to compare WT vs. hyh ovarian homogenates; (ii) since we observed evident age-related changes in GAPDH levels, anti-β-tubulin (AbCam, ab6046) was used to compare WT ovarian homogenates at different developmental stages; and (iii) since we wanted to compare equivalent number of cells, we decided to use a nuclear protein instead of a cytosolic protein to compare GC and ovarian tissue remnant extracts; thus, anti-histone H3 (Active Motif, 39163) was used in those experiments.

Recombinant proteins

For the generation of α-SNAP constructs, the plasmid pcDNA3.1 encoding α-SNAP WT and the plasmid pET28a encoding α-SNAP M105I (kindly provided by Dr. Phillys Hanson, Washington University, St. Louis, Missouri, USA) were used as templates. The coding sequence of full-length α-SNAP WT or α-SNAP M105I was obtained by PCR amplification and cloned in-frame into the *Eco*RI and *Sal*I sites of the pGST-Parallel-1 vector (91), and the nucleotide sequence of both recombinant constructs was confirmed by dideoxy sequencing. Expression and purification of the recombinant α-SNAP proteins, tagged with N-terminal glutathione *S*-transferase (GST) followed by a tobacco etch virus (TEV) protease cleavage site, were performed using similar methods described previously (92), with minor modifications. Briefly, expression in *E. coli* B834(DE3) (Novagen) was induced with 0.3 mM IPTG at 20° for 24 hours. Pellets of bacteria were suspended in homogenization buffer (50 mM Tris HCl, 0.5 M NaCl, 5 mM β-mercaptoethanol, 2 mM phenylmethylsulfonyl fluoride, pH 8.0), and lysed by sonication. The clarified supernatant was purified on glutathione-Sepharose 4B (GE Healthcare). After removal of the GST moiety by TEV cleavage, and sequential passage through glutathione-Sepharose 4B and Ni-NTA (QIAGEN) resins, α-SNAP proteins were further purified on a Superdex 200 column (GE Healthcare). Final purification was assessed by SDS-PAGE followed by SimplyBlue™ staining

(ThermoFisher).

Morphometric analysis of ovarian follicles

Serial sections (5 μm) of paraffin-embedded WT and hyh ovaries were mounted on slides and routine hematoxylin and eosin staining was performed for histologic examination by light microscopy. The number of follicles in each ovary was counted in every 10th serial section (i.e., 50 μm intervals). The entire ovary was analyzed but only follicles with visible oocyte nuclei were considered. Follicles were classified as primordial (oocytes surrounded by a single layer of flattened GCs), primary (oocytes surrounded by a single layer of cuboidal GCs and theca cells), preantral (oocytes surrounded by two or more layers of GCs with no antrum), early antral (non-confluent spaces between GCs) or antral (antrum within the GCs enclosing the oocyte). The total number of follicles and the number of each follicle type per ovary was determined. In addition, sections from were examined for the presence of corpora lutea. Atretic follicles were identified according to (52, 53). The presence of 5-20% pyknotic nuclei in the mural GC layer or in the antrum was used to define early stages of atretic follicles. Late stages of atresia were defined by follicular shrinkage and additional changes in the oocyte, which shows signs of resumption of meiosis, such as break down of the nuclear membrane with or without formation of a pseudo-maturation spindle, and oocyte fragmentation (52, 53).

Gonadotropic (superovulation) treatment and oocyte collection

Basically, 60 to 120 days old female WT and hyh mice were subjected to intraperitoneal injections of 5 IU of pregnant mare serum gonadotropin (PMSG; Syntex S.A., Argentina) followed 48 h later by 5 IU of human chorionic gonadotropin (hCG; Syntex S.A., Argentina). Since it has been demonstrated that response to exogenous PMSG is lowest among females in stages other than diestrus (87), PMSG was administered only in diestrus stage. Mice were maintained in a light-dark cycle from 8.00-20.00-8.00, i.e. light and dark periods last 12 hours.

Three different experiments were performed. In those experiments where ovaries were collected after 7h, 8h, and 9h post-hCG (see below, experiments A and C),

gonadotropin were administrated at 14.00 (light period). In those experiments where ovaries were collected after 13h post-hCG (experiment B, oocyte collection), gonadotropins were injected at 19.00 (light period).

(A) Ovaries were collected at 0 h (pre-PMSG injection or no treatment), and 7 h, 8h and 9 h after hCG injection. Four animals of each genotype (WT and hyh) were included in each time point. Mutant hyh females were analyzed only at 0h (no treatment) and 8 h after hCG injection. Thus, the ovaries of 16 WT females and 8 hyh females subjected to gonadotropic stimuli were analyzed as follows: (i) one ovary from each of four animals was collected and stored at -80ºC for later preparation of protein homogenates and Western blot analysis (performed in duplicate), and (ii) the other ovary of each animal was processed for light microscopy and morphometric analysis (see above).

(B) Another group of 31 WT females and 19 hyh females were subjected to the same superovulation treatment, and 13 h after hCG injection, Metaphase II oocytes were collected from the oviductal ampulla into Earle's balanced salt solution with 0.01% PVA, 0,001% Gentamycin, and 25 mM Hepes buffer, pH 7.3 (MEM/HEPES) and counted under a microscope, as described previously (17). Four animals from each group were randomly selected and processed for light microscopy to quantify the presence of corpora lutea.

(C) A group of 3 P60 WT females and 3 P60 hyh mutant females were subjected to the same superovulation treatment, and 8 h after hCG injection, ovaries were removed and processed for purified GC isolation and protein extraction (see above). Three P60 WT females and three P60 hyh mutant females that received no treatment were used as controls.

Isolation of Cumulus-Oocyte Complexes (COCs)

COCs were collected from antral follicles. To this end, females were primed by intraperitoneal (i.p.) injections with 10 IU of PMSG (Syntex, Argentina), and 45–48 h later COCs were obtained by puncturing ovarian antral follicles. The collection medium was Earle's balanced salt solution with 0.01% PVA, 0,001% Gentamycin, and 25 mM Hepes buffer, pH 7.3 (MEM/HEPES) supplemented with 2.5 μM Milrinone to

inhibit oocyte maturation. No denudation of cumulus cells was performed to use COCs. Only GV oocytes (about 80 μm in diameter) with intact cumulus (more than 5 GCs layers) were used. COCs were cultured until use in drops of CZB medium (EmbryoMax® CZB, Merck Millipore) under mineral oil at 37°C in a humidified atmosphere of 5% CO2 in air.

Terminal deoxynucleotidyl transferase dUTP nick end labeling (TUNEL) assay

Apoptosis of GCs (tissue samples) and cumulus cells (COCs) was evaluated using In situ Cell Death Detection Kit, Fluorescein (Roche Molecular Biochemicals, Germany) according to the manufacturer's instructions. Ovary samples were fixed in Bouin´s solution and permeabilized in 0.1M Tris-HCl (pH7.5) containing 3% BSA and 20% bovine fetal serum. COCs were fixed in 3,7% paraformaldehyde in PBS, and washed three times in PBS containing 1% BSA (PBS/BSA). The fixed COCs were permeabilized in 0.1% Triton X-100 in 0.1% sodium citrate for 1 hour at room temperature and washed three times in PBS/BSA. The samples were then incubated with TUNEL reaction mixture for 1 hour at 37°C in a dark and damp place. After washing, samples were mounted in Vectashield Mounting Medium (Vector Laboratories, Burlingame, CA) with slight coverslip compression, sealed, and stored at 4°C until visualization. In COCs samples 1 μg/ml *propidium iodide* (Molecular Probes, Invitrogen) was added for DNA detection. Slides were analyzed using a laser-scanning confocal microscope (Olympus Fluoview FV1000). For each experimental series, images were captured using the same microscope settings. ImageJ software (version 1.42l; NIH, MD) was used for the analysis of the images. Apoptosis was expressed as (i) the number of TUNEL positive cells or bodies per area, or (ii) the percentage of TUNEL positive nuclei per COC. In ovarian tissue samples, 3 sections per ovary were analyzed (n=3 animals per genotype). All follicles in a given section were pictured and a grid of non-overlapping fields of 0.032 mm^2 (200μm.160μm) was applied to each follicle. Fields completely included within a follicle were considered for quantification; thus, only TUNEL+ GCs were examined. When the field was 100% in the mural GC layers, 70-80 cells were approximately contained. In the case of antral follicles, fields containing antrum + mural GCs were also considered. At least 5 fields (0.16mm^2) were analyzed

per section but in most cases more than 10 fields per section were quantified. Since many TUNEL+ cells in hyh mutant follicles are found in the antrum or in the GC layers adjacent to the antrum, the number of TUNEL+ cells was normalized per area and not per total cells. Negative and positive controls of the TUNEL reaction were included in each experimental set. In the negative controls, samples were incubated with label solution (without terminal transferase) instead of TUNEL reaction mixture. For positive controls, samples were incubated with recombinant DNase I (5U/ml) *(Omega Bio-Tek*, Norcross, GA, USA) for 10 min at RT to induce DNA strand breaks, prior to labeling procedures.

Determination of estrous cycle stage by vaginal cytology

A vaginal swab was collected using a cotton-tipped swab wetted with physiological saline solution at ambient temperature and inserted into the vagina of the restrained mouse. Cells were transferred to a dry glass slide by rolling the swab across the slide, overlaid with a coverslip, and viewed immediately at 40× magnification under bright field illumination.

Mice mating system

Mice (8 to12 weeks old) were genotyped and mated using the monogamous mating system (i.e., an individual male and an individual female mouse were introduced the same cage for mating and were kept together until the end of the experiment). Vaginal plug was used as an indication that mating had occurred. If no litters were produced after 60 days from (i) the starting experimental day or (ii) the last litter, the experiment was finished. In the former situation, the mating was considered non-productive. The number of productive matings (litters) per female and the number of pups per litter were counted in each mating pair.

Data Analysis

Statistical analysis was determined using Student's t-test for individual comparisons or ANOVA test for multiple comparisons. Data are expressed as means ± SEM, and

differences were considered significant when $p<0.05$. The statistical analysis and the graph plots were done using GraphPad Prism software.

Supplementary Figures

α-SNAP is expressed in mouse ovarian granulosa cells and plays a key role in folliculogenesis and female fertility.

Alexis Arcos, Matilde de Paola, Diego Gianetti, Diego Acuña, Zahady D. Velásquez, María Paz Miró, Gabriela Toro, Bryan Hinrichsen, Rosa Iris Muñoz, Yimo Lin, Gonzalo A. Mardones, Pamela Ehrenfeld, Francisco J. Rivera, Marcela A. Michaut, Luis Federico Batiz

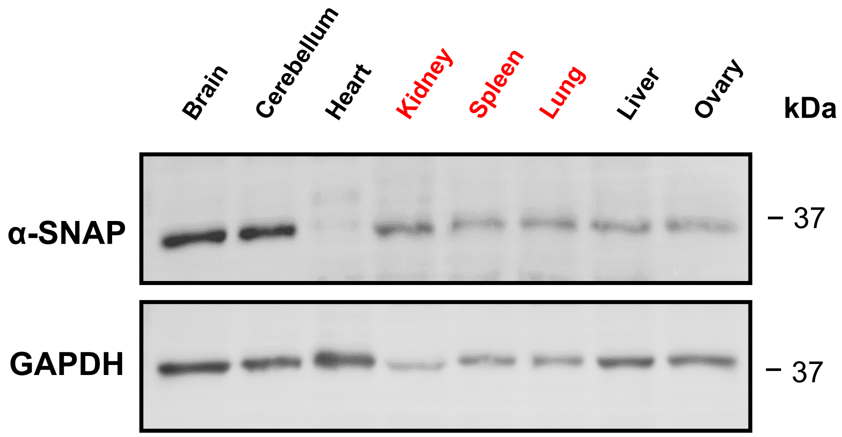

Figure S1. α-SNAP expression in different tissues of P60 wild type (WT) female mouse. Full-length Western blots corresponding to Figure 1A (main article) showing α-SNAP protein levels in different postpubertal (postnatal day 60; P60) WT female tissue extracts. GAPDH levels serve as loading control. Tissue extracts showing consistently lower levels of GAPDH compared to ovarian extracts are shown in red and were not included in Figure 1A (main article).

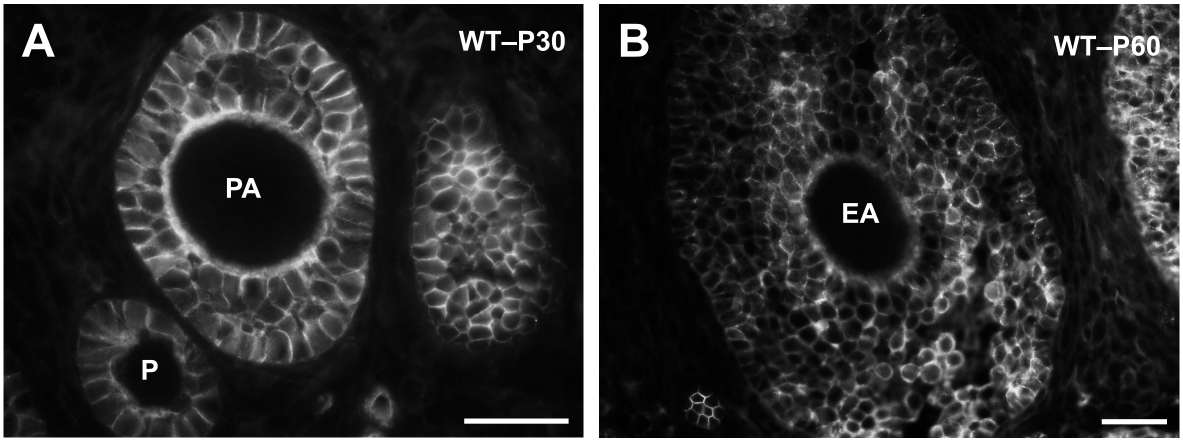

Figure S2. Immunofluorescence staining for N-cadherin in ovary sections from P30 (**A**) and P60 (**B**) WT females. N-cadherin protein is highly and preferentially expressed in GCs during folliculogenesis. P: primary follicle, PA: preantral follicle, EA: early antral follicle. Scale bars, 20μm**.**

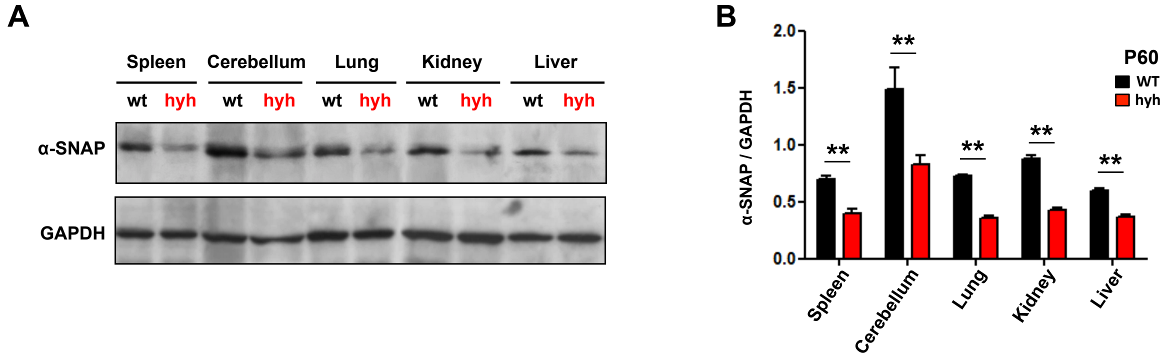

Figure S3. α-SNAP protein levels in tissue extracts from wild type (WT) and α-SNAP mutant (hyh) females. A. Western blot analysis of α-SNAP in postpubertal (postnatal day 60; P60) WT female mouse tissue extracts. GAPDH levels serve as loading control. **B.** Quantification of Western blots. Bars represent mean ± SEM of densitometric analyses (n = 3 independent experiments). * $p<0.05$; ** $p<0.01$ (Student's t-test).

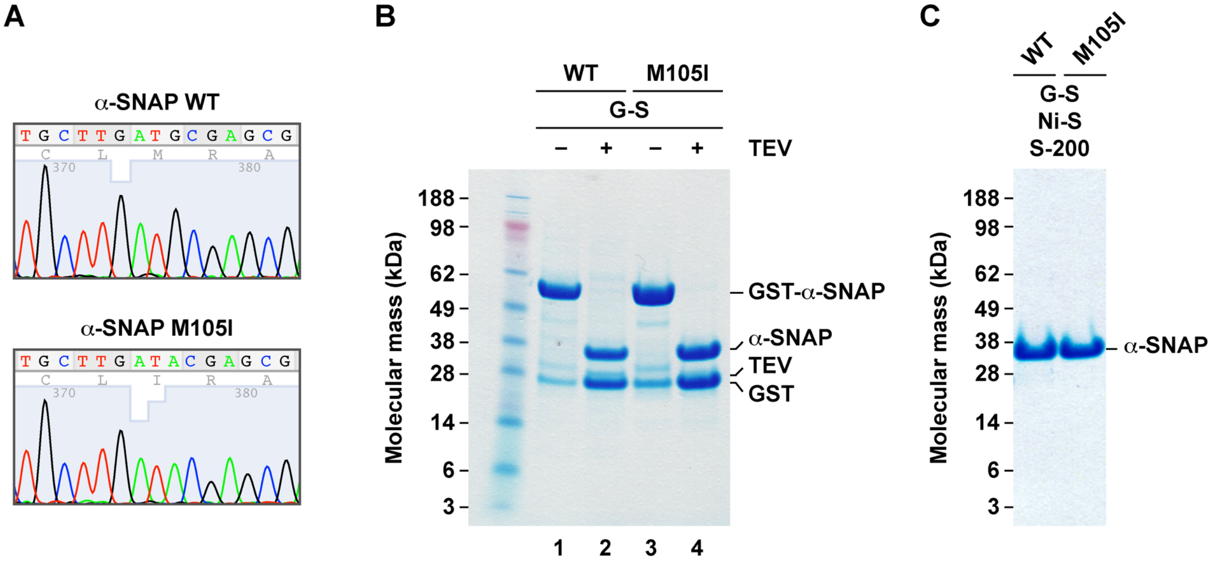

Figure S4. Purification of recombinant bovine α-SNAP proteins. A. DNA sequence of the plasmid pGST-Parallel-1 encoding either full-length α-SNAP WT or full-length α-SNAP M105I tagged with N-terminal glutathione *S*-transferase (GST) followed by a tobacco etch virus (TEV) protease cleavage site. **B.** Cultures of *E. coli* B834(DE3) transformed with the plasmid encoding either GST-α-SNAP WT or GST-α-SNAP M105I were incubated with 0.3 mM IPTG at 20° for 24 hours. After bacteria homogenization, GST-fusion proteins were enriched by affinity chromatography on glutathione-Sepharose 4B (*G-S*; lanes 1 and 3), followed by treatment with His-tagged TEV protease to cleave the GST moiety (lanes 2 and 4). **C.** GST-fusion proteins treated with His-tagged TEV protease were subjected to sequential passage through glutathione-Sepharose 4B (*G-S*) and Ni-NTA (*Ni-S*) resins to remove the GST moiety and the His-tagged TEV protease, respectively, and α-SNAP proteins were further purified on a Superdex 200 column (*S-200*). **B-C.** Proteins were analyzed by SDS-PAGE in 4-12% gradient gels, and stained with SimplyBlue™. The position of molecular mass markers is indicated on the left.

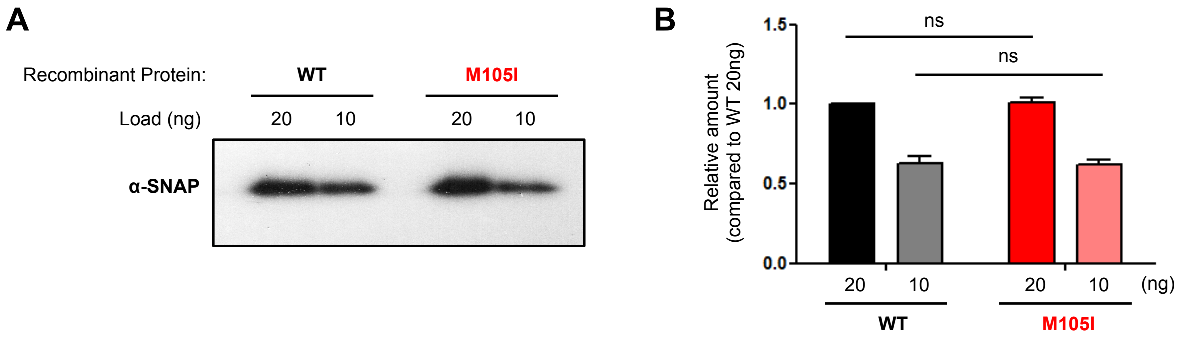

Figure S5. Test of anti-α-SNAP immunoreactivity. A. Western blot of α-SNAP loading WT and M105I recombinant proteins. 20 or 10 ng of purified recombinant protein were used per lane. **B.** Quantification of Western blots. Bars represent mean ± SEM of relative densitometric analyses. WT recombinant protein (20ng) was used as reference (n=3 independent experiments). ns, non-significant difference (Student's t-test).

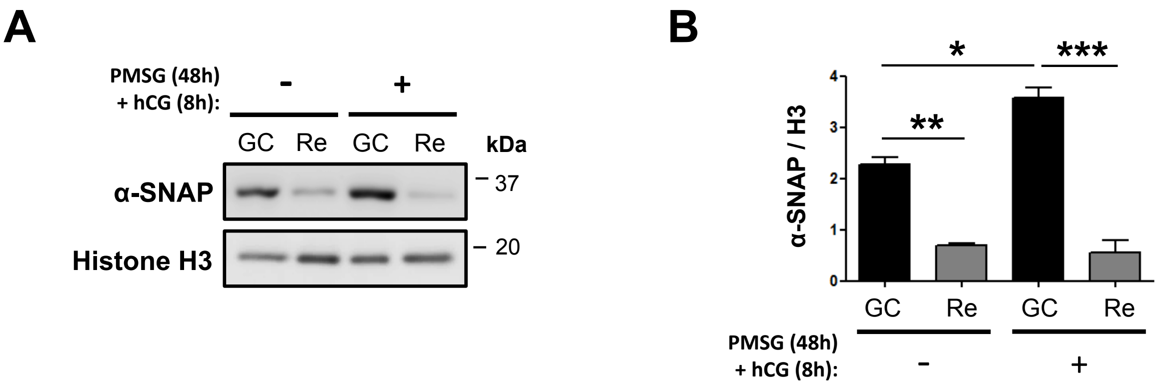

Figure S6. **A.** Western blot of α-SNAP in proteins extracts of purified granulosa cells (GC) and ovary remnants depleted of GC (Re) obtained from (i) non-treated WT P60 females, and (ii) WT P60 females subjected to superovulation (PMSG+hCG) treatment (ovaries were removed 8h after the hCG injection). Histone H3 was used as loading control. **B.** Quantification of Western blots. Bars represent mean ± SEM of densitometric analyses (n=3 independent experiments). * $p<0.05$; ** $p<0.01$; *** $p<0.001$ (ANOVA with Tukey's post hoc test).

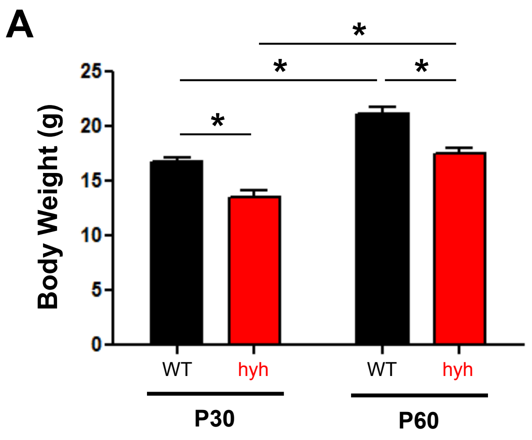

Figure S7. A. Body weight in grams (g) of WT and hyh females at P30 and P60 at the moment of ovary extraction. **B.** Ovary weight in milligrams (mg). Both ovaries were weighed and the mean was considered the value of ovary weight for each female. **A-B.** Bars represent mean ± SEM of 4 independent experiments. * $p<0.05$ (ANOVA).

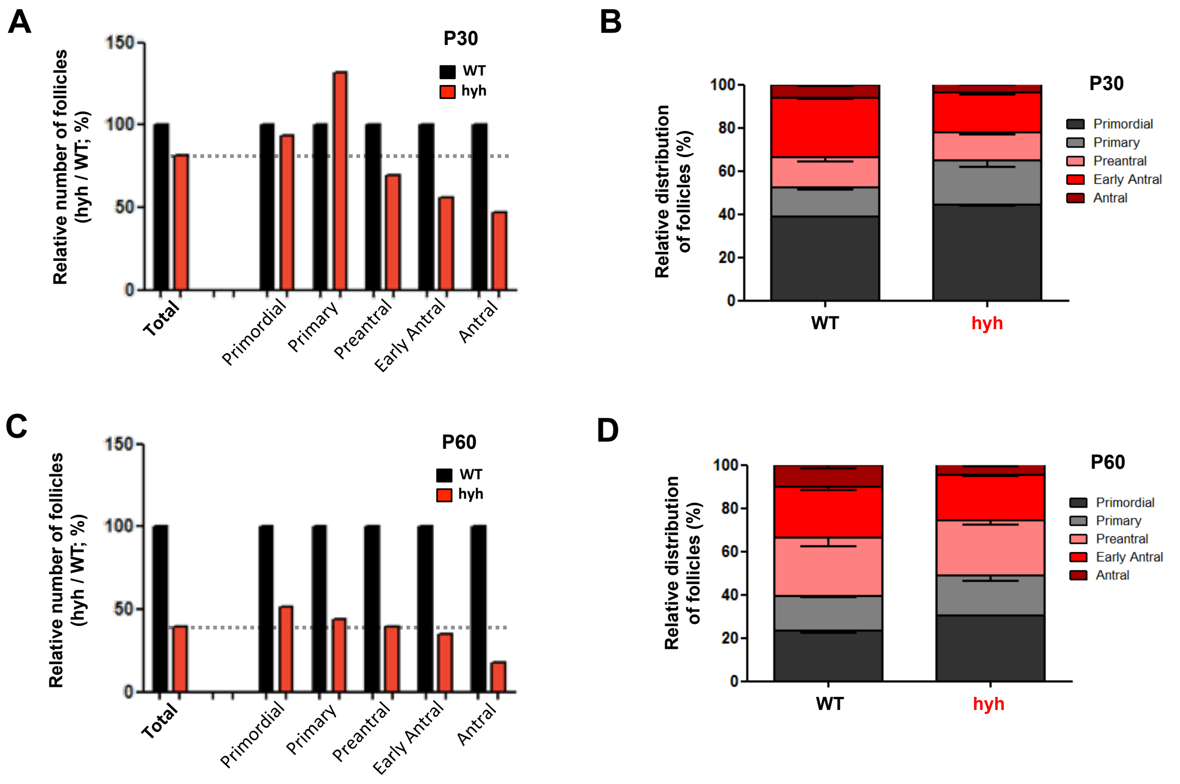

Figure S8. A, C. Relative number (%) of follicles in hyh compared with WT ovaries at P30 **(A)** and P60 **(C)**. The total number of follicles (Total) and the number of follicles at different stages (from primordial to antral) were analyzed. **B, D.** Relative distribution (%) of follicle types within WT and hyh ovaries at P30 **(B)** and P60 **(D)**. Bars represent the frequency distribution (cumulative percentage) of different follicle types in each condition.

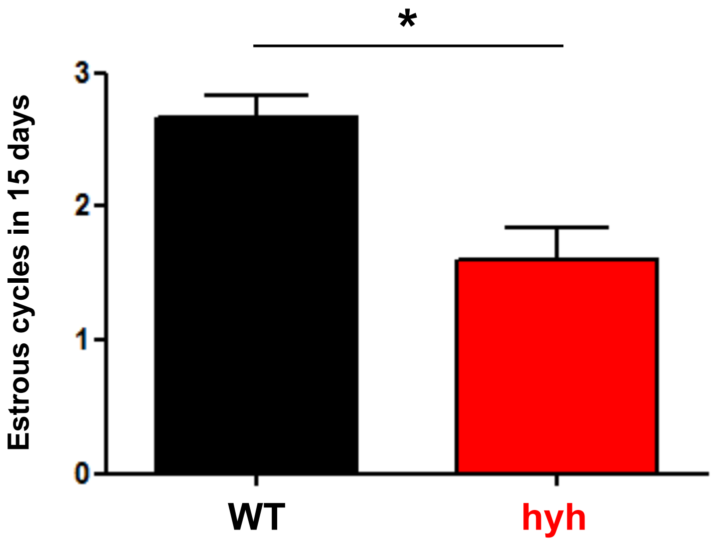

Figure S9. Number of estrous cycles in a 15-days period. Bars represent mean ± SEM of 10 (WT) and 5 (hyh) independent experiments. * $p<0.05$ (Student´s t-test).

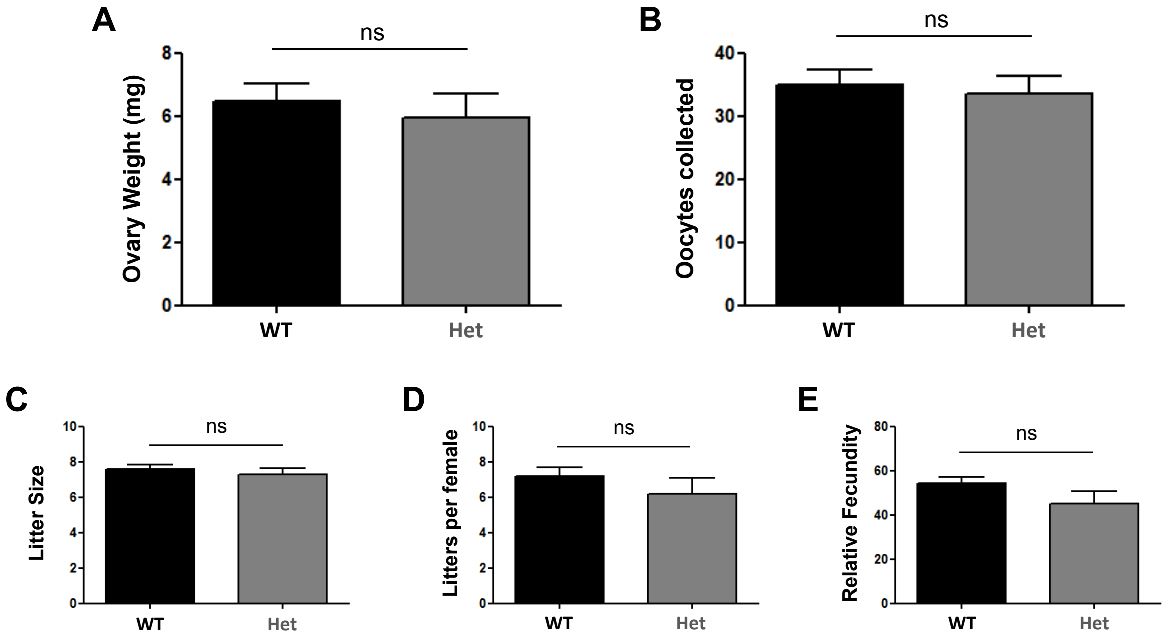

Figure S10. Comparison of ovarian phenotype parameters and reproductive performance between WT and hyh heterozygous (Het) females. **A.** Ovary weight of non-stimulated females in milligrams (mg). **B.** Quantification of MII oocytes collected from the oviductal ampulla 13h after gonadotropic (PMSG x 48h + hCG) stimulation. Bars represent mean ± SEM (n=31 WT and 11 Het). **C-E.** Quantification of the litter size (**C**), number of litters per female (**D**), and relative fecundity (**E**). Relative fecundity is obtained as: (litter size) x (number of litters) x (productive matings/100); the value obtained is a measure of the overall fecundity according to the Handbook of Genetically Standardized JAX Mice. Bars represent mean ± SEM (n=10 WT and 10 Het). ns, non-significant difference (Student's t-test).
